# Decoupled Asian monsoon intensity and precipitation during glacial-interglacial transitions on the Chinese Loess Plateau

**DOI:** 10.1038/s41467-022-33105-2

**Published:** 2022-09-14

**Authors:** Yukun Zheng, Hongyan Liu, Huan Yang, Hongya Wang, Wenjie Zhao, Zeyu Zhang, Miao Huang, Weihang Liu

**Affiliations:** 1 College of Urban and Environmental Sciences and MOE Laboratory for Earth Surface Processes, Peking University, Beijing, China; 2 Hubei Key Laboratory of Critical Zone Evolution, School of Geography and Information Engineering, China University of Geosciences, 430074 Wuhan, China; 3 State Key Laboratory of Biogeology and Environmental Geology, School of Earth Sciences, China University of Geosciences, 430074 Wuhan, China; 4 Academy of Disaster Reduction and Emergency Management, Faculty of Geographical Science, Beijing Normal University, 100875 Beijing, China

**Keywords:** Palaeoclimate, Climate-change ecology

## Abstract

The discrepancies among the variations in global ice volume, cave stalagmite δ^18^O and rainfall reconstructed by cosmogenic ^10^Be tremendously restrain our understanding of the evolution of the East Asian summer monsoon (EASM). Here, we present a 430-ka EASM mean annual precipitation record on the Chinese Loess Plateau obtained using branched glycerol dialkyl glycerol tetraethers based on a deep learning neural network; this rainfall record corresponds well with cave-derived δ^18^O data from southern China but differs from precipitation reconstructed by ^10^Be. Both branched tetraether membrane lipids and cave δ^18^O may be affected by soil moisture and atmospheric temperature when glacial and interglacial conditions alternated and were thus decoupled from atmospheric precipitation; instead, they represent variations in the intensity of the EASM. Furthermore, we demonstrate that the brGDGT-DLNN method can significantly extend the temporal scale record of the EASM and is not restricted by geographic location compared with stalagmite records.

## Abstract (web summary)

In considering Asian monsoon intensity and precipitation during glacial-interglacial transitions in Chinese Loess Plateau, a new study finds that brGDGT-DLNN method can significantly extend the temporal scale record of the EASM and is not restricted by geographic location compared with stalagmite records.

## Introduction

The loess–paleosol sequences (LPSs) on the Chinese Loess Plateau (CLP), recognized by Heller & Liu^1^, contain a significant climatic archive and precise chronological controls for terrestrial paleoclimatic research. The chronology of loess records spanning the past three glacial–interglacial cycles in China has been dramatically improved in current years by using newly developed K-feldspar luminescence dating^2^. In recent decades, records of the East Asian summer monsoon (EASM) in LPSs and speleothems have considerably enhanced our comprehension of its evolutionary history and dynamic mechanisms^3,4^. It has been consistently accepted that the Chinese LPSs correlates well with benthic foraminiferal δ^18^O records, which can reflect the variations in sea level and ice-volume cycles^5^. The EASM reconstructed by LPSs corresponds well with the high-latitude ice volume in the Northern Hemisphere, which shows a 100-ka dominant cycle after the Middle Pleistocene^6,7^. Although several proxies based on LPSs indicate concomitant 100-ka and 23-ka periods (i.e., loess ^10^Be^8^ and δ^13^C_IC_^9^), a few new proxies display no 23-ka cycle (i.e., microcodium Sr/Ca^10^ and East China Sea local seawater δ^18^O^11^). These factors all restrain our understanding of the evolution of the EASM and, more importantly, its response to ice-volume forcing and insolation. Another significant proxy for the EASM, stable oxygen isotopes of speleothems in caves from southern China with robust chronological controls^12^, displays a dominant precessional period (23 ka), which indicates that the EASM is directly controlled by summer insolation in the Northern Hemisphere^13,14^. However, the interpretations of speleothem δ^18^O are controversial^15^ due to, for example, regional rainfall, the seasonality of precipitation over the cave site^4^, and the outwind rainout of air masses^16^.

Some studies have attempted to reconstruct the paleoclimate, including the mean annual temperature (MAT) and mean annual precipitation (MAP), on the CLP. Phytoliths were used in the early years of research as a proxy to reconstruct MAT and MAP on the CLP^17,18^. However, the low classification resolution of phytoliths may expand their tolerance to climatic factors and affect their sensitivity to environmental variations^17^. Another proxy, the carbon isotopic composition of bulk soil organic matter (δ^13^C_SOM_), reflects the relative abundances of C_3_ and C_4_ plants for reconstructing MAP on the CLP^19^. However, this proxy is also affected by temperature, making it difficult to tease apart the precipitation signal^20^. Afterward, the utilization of cosmogenic ^10^Be, which is generated in the atmosphere and readily adsorbed by aerosols and then carried to surface sediments with precipitation, made it possible to quantitatively reconstruct MAP on the CLP, revealing the low-latitude interhemispheric insolation-driven EASM intensity^8^.

One promising geochemical proxy, branched glycerol dialkyl glycerol tetraethers (brGDGTs) (Supplementary Fig. 1), was confirmed to have a bacterial origin^21^, and a vast number of studies have investigated the environmental controls on this proxy. The study sites where brGDGTs have been used cover a great part of the world, and brGDGTs lipids can persist in the environment up to at least 55 million years ago^22^. Previous empirical observations have revealed that the methylation and cyclization degree of brGDGTs have tight connections with MAT and soil pH, respectively^23–26^. More specifically, many studies have reconstructed paleotemperature and paleosol pH changes at various temporal and spatial scales globally based on these relationships^27–29^. Three structural varieties of brGDGTs, containing an uncharacterized isomer of brGDGT Ic (Supplementary Fig. 1), have been confirmed in an Acidobacteria species that was cultured under oxygen limitation^30^. Nevertheless, it has been difficult to trace the exact bacterial sources of the other brGDGTs, and subsequent studies have revealed that brGDGTs may be derived from aquatic environments^31^. Recently, some studies have attempted to use various statistical approaches to improve the correlation coefficients between brGDGTs proxies and climate data by setting up nonlinear relationships. For example, Crampton-Flood and coauthors utilized the Bayes model to enhance the precision of MAT estimation based on brGDGTs^32^. Moreover, Martinez-Sosa et al.^33^ and Ragberg et al.^34^ also used nonlinear approaches to calibrate brGDGTs in lakes to temperature, enhancing the precision of models in predicting terrestrial surface temperatures. In addition to temperature, other factors, MAP, soil water content and bacterial community structure, were found to affect brGDGTs based proxies^35^. Although brGDGTs definitely have bacterial origins, empirical studies have found that the soil water content (SWC) may affect the methylation degree of brGDGTs, and 6-methyl brGDGTs isomers are significantly affected by SWC, especially in arid and semiarid regions^36^. When the relative amount of 6-methyl vs. 5-methyl brGDGTs (IR_6ME_) >0.5, one promising index, MBT’, which expresses the methylation degree of brGDGTs, can potentially represent variations in SWC. However, it is unprecedented for brGDGTs to forecast MAP based on established quantitative transfer functions. In addition, the temperature reconstructed by brGDGTs on the CLP does not correspond well with glacial–interglacial cycles^27^, and discrepancies are also observed between the rainfall amounts reconstructed by ^10^Be and Chinese cave δ^18^O values^8^.

**Fig. 1:**
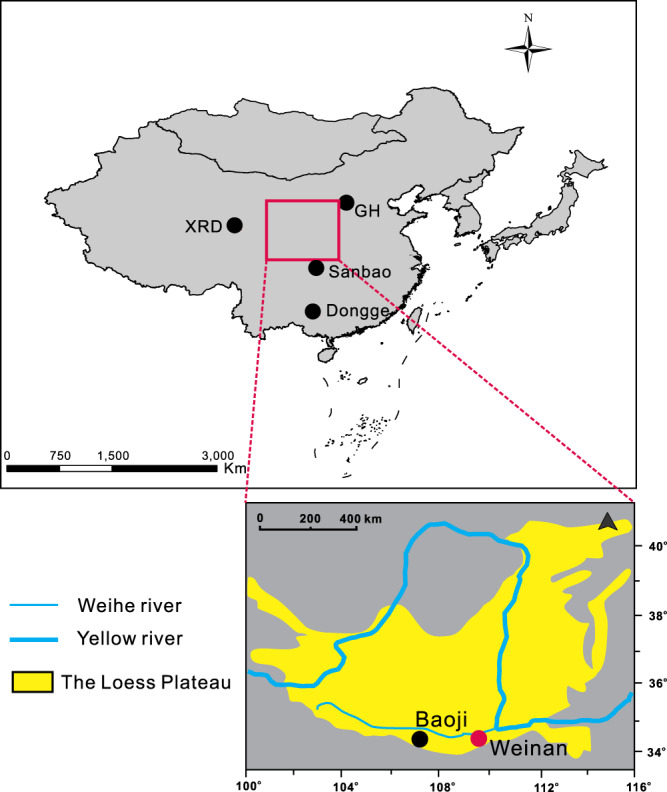
Geographic locations of Weinan and Baoji loess–paleosol profiles on the Chinese Loess Plateau. And the locations of study sites mentioned in this text include: Sanbao cave^8^, Xiangride (XRD)^55^, Gonghai (GH)^56^, and Dongge cave^4^. The base map was generated using ArcGIS software.

In this study, we established an optimal MAP model based on all 6-methyl brGDGTs, Ia and Ic, and applied this method to a well-dated loess–paleosol profile in Weinan (430 ka before present (BP), Fig. 1) covering five glacial–interglacial periods. Combined with global terrestrial brGDGT-calibrated temperature based on some of the 5-methyl brGDGTs isomers and soil pH models based on some of the 6-methyl brGDGTs isomers established by De Jonge and colleagues^24^, we reconstructed the variations in MAT and paleo-pH after 430 ka BP in Weinan profile. Furthermore, using a promising SWC proxy, MBT’, we obtained the relative SWC changes in the derived profile. We hypothesized that the brGDGT-based mean annual precipitation (MAPc) might deviate from the actual atmospheric precipitation, different from that reconstructed by ^10^Be from the atmosphere. Instead, the records of brGDGTs may indeed reveal a complicated variation in the EASM that contains the temperature or SWC signal.

## Results

### brGDGT-MAP models

Here, we used 712 previously published brGDGTs surface soil samples from around the world (Supplementary Figs. 2 and 3) and indicated the distributions of various brGDGTs compounds. In addition, the MAP data of each soil site applied in this study were all published in previous studies (Table 1). Through analyses of the linear relationships between the fractional abundances (FAs) and MAP obtained from various brGDGTs compounds, we found that only a few brGDGTs compounds correlated well with MAP: Ia (Supplementary Fig. 4a) was 0.55, IIa’ (Supplementary Fig. 4e) was 0.34, and IIIa’ (Supplementary Fig. 4k) was 0.30. The majority of brGDGTs isomers had low *R*^2^ values, indicating that they have little direct correlation with MAP. In particular, the *R*^2^ values for the correlations between the FAs of Ib, IIa, IIc, and MAP were 4.56 × 10^-4^, 0.003, and 0.007, respectively (Supplementary Fig. 4b, d, h).

**Table 1:** List of global surface soil datasets

Reference	Quantity of surface soils	brGDGTs compound types	DOI
De Jonge et al.^24^	191	15	10.1016/j.gca.2014.06.013
Xiao et al.^58^	27	15	10.1016/j.orggeochem.2015.10.005
Yang et al.^59^	26	15	10.1016/j.orggeochem.2015.02.003
Lei et al.^60^	44	15	10.1016/j.orggeochem.2016.02.003
Wang et al.^61^	93	15	10.1016/j.quascirev.2020.106172
Wang et al.^62^	56	15	10.1016/j.orggeochem.2016.05.013
Ding et al.^63^	27	15	10.5194/bg-12-3141-2015
Naafs et al.^23^	96	15	10.1016/j.orggeochem.2017.01.009
Crampton-Flood et al.^32^	77	15	10.1016/j.gca.2019.09.043
Wang et al.^64^	28	15	10.1038/s41598-017-17964-0
Wang et al.^65^	47	15	10.1038/s41598-019-39147-9

We then selected 533 surface soil samples as the training dataset and 179 surface soil samples as the validation dataset, both of which satisfied the principle of randomness. We assessed the precision of the model using the values of the validation dataset’s *R*^2^ and root mean square error (RMSE) values. By means of several parameter tests in brGDGT-MAP models (Supplementary Figs. 5–9), our deep-learning neural network (DLNN) methods could precisely capture the multidimensional and nonlinear relationships between the brGDGTs isomers and MAP Supplementary Fig. 10, for training dataset: *R*^2^ = 0.82, RMSE = 291 mm, and *n* = 533; for the validation dataset: *R*^2^ = 0.80, RMSE = 287 mm, and *n* = 179). As a result, brGDGTs can serve as optimal tools for reconstructing global MAP variations, especially considering their global ubiquity.

### Paleoclimate and paleoenvironment reconstruction in Weinan

**Fig. 2:**
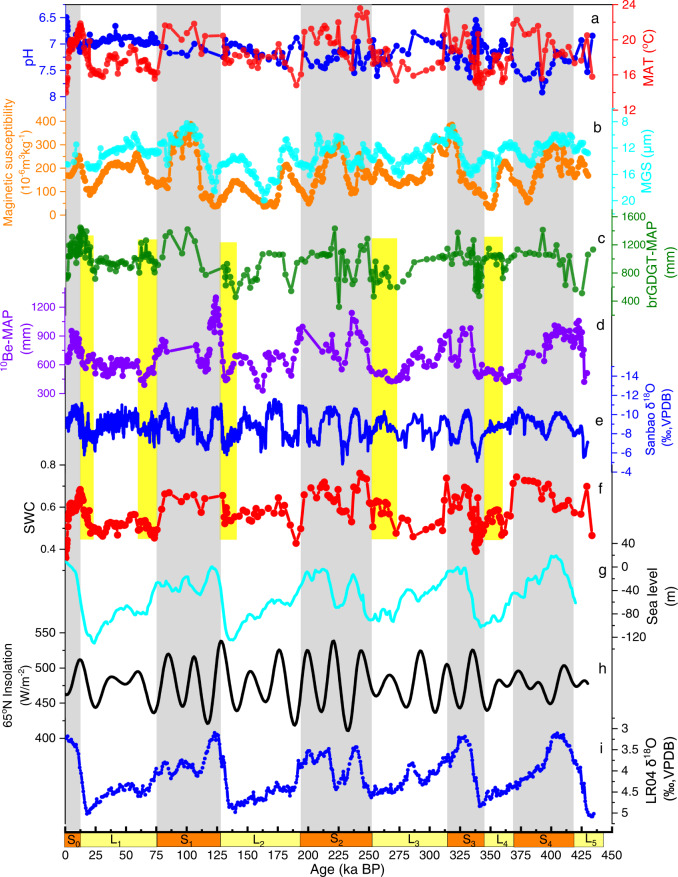
Variations in brGDGTs parameters compared with the parameters of other records. **a** The pH calibration based on 5- and 6-methyl branched glycerol dialkyl glycerol tetraethers (brGDGTs, dark blue solid line, this study) and mean annual average temperature (MAT) calibration based on 5- and 6-methyl brGDGTs (dark red solid line, this study). **b** The mean grain size (mm) (MGS, blue solid line, this study) and magnetic susceptibility (orange solid line, this study). **c** The mean annual average precipitation (MAPc) values reconstructed by the brGDGT-MAP model (Weinan, dark green solid line, this study). **d** The MAP values reconstructed by ^10^Be (Baoji, purple solid line). **e** The variations in Sanbao δ^18^O (the dark blue solid line indicates the results of the five-point average). **f** The changes in the MBT’ (dark red solid line), representing the soil water content (SWC) variations^36^. **g** The changes in sea level (blue solid line)^57^. **h** The solar radiation at 65°N (black solid line)^42^. **i** The benthic foraminifera-derived δ ^18^O stack (dark blue solid line)^41^. The orange and yellow bars represent the loess–paleosol cycles in the Weinan profile. The gray shading bars represent the interglacial periods in the Weinan profile, and the light-yellow bars highlight the decoupled periods between the ^10^Be reconstructions and our results.

**Fig. 3:**
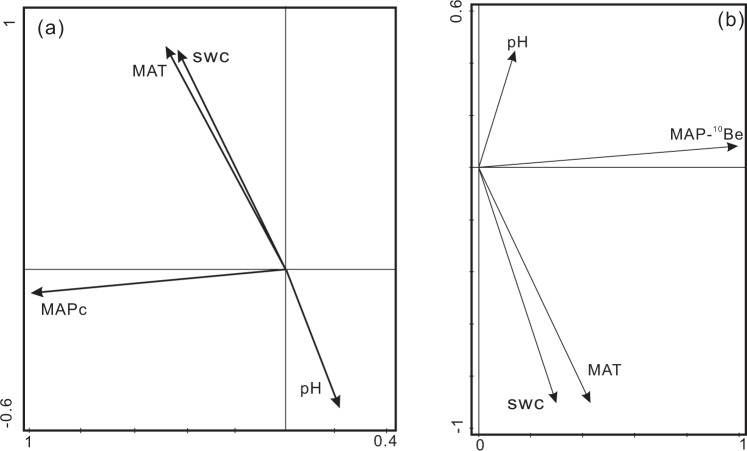
Principal component analysis (PCA) among various environmental factors on the Chinese Loess Plateau. **a** brGDGT-MAP (mean annual average precipitation), MAT (mean annual average temperature), SWC (soil water content), and pH in Weinan since 430 ka BP (*n* = 136) and **b** ^10^Be-MAP, MAT, SWC and pH when glacial and interglacial periods alternated (*n* = 77). Totals of 98% (**a**) and 90% (**b**) of the variations in the paleoclimate data were explained by the first two PCA axes.

In combination with the age-depth model obtained by interpolation between geomagnetic polarity boundaries and using magnetic susceptibility as an indicator of accumulation rate, as well as the U–^230^Th-dated oxygen isotope records from Sanbao caves in central China (Supplementary Fig. 15), paleotemperature records reconstructed from brGDGTs in the Weinan loess–paleosol profile after 430 ka BP (Fig. 2a) could aid in further interpreting the timing of glacial terminations in northern China. Indeed, based on multiyear observed temperature records at the nearest meteorological station, the modern mean annual temperature in Weinan was 13.8 °C, which is similar to the brGDGT-inferred temperature at the top of S_0_. Moreover, our 430-ka MAT records clearly show the dominance of the 100-kyr cycles, which correspond well with the loess–paleosol cycles and reveal the variations (in a range of ∼10 °C) in MAT on glacial–interglacial timescales. The magnetic susceptibility and mean grain-size (MGS) records also display a majority cycle of 100 ka (Fig. 3b).

Regarding the soil paleo-pH, the temperature changes were almost opposite to the variations in soil pH over the last 430 ka BP (Fig. 2a). Similar to the MAT reconstruction, the dominant soil pH cycle also showed a 100-kyr period and revealed apparent variations (within a range of ∼0.5) in pH on glacial–interglacial timescales. We found that the pH value in S_4_ reached its lowest level at 430 ka, and the soil was acidic for a long time. The results showed that the minimum and maximum pH values recorded over the last 430 ka were 6.1 (∼380 ka) and 7.6 (∼0.2 ka), respectively. The most extensive pH range in the five interglacial periods (S_0_–S_4_) was ∼1.1 that was found in S_4_.

To obtain the MAP reconstructions, we entered all 6-methyl brGDGTs, Ia and Ic samples into the brGDGT-MAP model, which may be suitable for reconstructions on the CLP, especially in semiarid and semihumid regions. As a result, we obtained the variations in MAPc in Weinan over the past 430 ka (Fig. 2c). We found an obvious glacial–interglacial cycle in this MAPc curve. The absolute value of MAPc in the Weinan profile was almost equal to the observed multiyear MAP in this region (2000–2015) when the samples approach the surface (S_0_).

With regard to SWC, we found that nearly all IR_6ME_ values, exceeded 0.5 in this profile (Supplementary Fig. 16). As a result, MBT’ can be regarded as a potential SWC indicator in the Weinan LPS. Here, we found a dominant 100-kyr cycle in the MBT’ record (Fig. 2f), representing relatively high SWC during interglacial periods and relatively low SWC values in glacial periods in 430 ka BP in the Weinan LPS.

## Discussion

With the brGDGT-MAP model we established and the paleoclimate we reconstructed in the Weinan profile over the past 430 ka, we can better understand the variation in EASM history in this region. Regarding the brGDGT-MAP model, we found that the 5-methyl and 6-methyl brGDGTs isomers display different linear correlations with MAP, and this difference may be due to the 5-methyl brGDGTs isomers containing a more significant temperature signal compared with the pH, soil moisture and MAP signals^24^. On the other hand, some studies have revealed that 6-methyl brGDGTs are more responsive to soil pH and may be more affected by soil moisture than other indicators, and these variables have close relationships with MAP^36^. More importantly, we reveal that the relationships between brGDGTs and modern MAP are not direct linear correlations. The adequate evidence we conclude is as follows: first, we entered the same arguments (all 6-methyl brGDGTs isomers, Ia and Ic) into the multiple linear regression model, and the results show the poor forecast ability both in the training dataset and validation dataset, especially when those soil-sites measured MAP > 1500 mm (Supplementary Figs. 5 and 6). Furthermore, we indicate that the larger linear R^2^ value between the 6-methyl isomer and MAP does not indicate a better capacity of this indicator to predict MAP. For example, although the linear *R*^2^ value of Ic and MAP is obviously larger than that of IIIc’ and MAP (Supplementary Fig. 4), the IIIc’ isomers are more critical than Ic in predicting the MAP values in our DLNN model (Supplementary Fig. 9). In addition to Ic and IIIc’ in our brGDGT-MAP model, the R^2^ values of the validation dataset decrease more when excluding IIIc’ than when excluding Ic. We further utilized a more complex artificial neural network (ANN) (i.e., recurrent neuron network (RNN), long short-term memory networks (LSTM) and gate recurrent unit (GRU)) to predict MAP based on brGDGTs (Supplementary Fig. 11)). The results also reveal that our DLNN model is the most suitable one to apply in this field (Supplementary Fig. 12). Finally, we tested this brGDGT-MAP model in the Xiangride (XRD) profile (Fig. 1), and the variations in the Holocene MAP we reconstructed correspond well with the most reliable rainfall proxies in the EASM region (Supplementary Fig. 14) and we concluded that brGDGTs can be used as a robust tool to quantitatively reconstruct MAP on a suborbital time scale.

A previous study found a robust relationship between water balance and soil pH^37^. The latter can be calculated well by 6-methyl brGDGTs. More recently, it was found that the distribution of Actinobacteria is positively correlated with aridity, maintaining the same trend as that of 6-methyl brGDGTs^38^. Indeed, our study further revealed the quantitative relationship between MAP and 6-methyl isomers through the DLNN model.

The MAPc we reconstructed differs from the reconstruction obtained from ^10^Be. Although quite a few studies have developed quantitative models between brGDGTs indices and MAT or soil pH values on global or regional scales, MAP is difficult to reconstruct in the Weinan LPS. Compared with reconstructions of temperature and soil pH, MAP reconstructions, especially on geologic timescales, are more difficult to obtain and lack reliable proxies. Through the brGDGT-MAP model, we obtained the MAPc changes over the past 430-ka BP (Supplementary Fig. 15)^39,40^. Another MAP reconstruction was obtained by an empirical cosmogenic proxy (^10^Be) in Baoji (CLP, China, Figs. 1 and 2d), located in northwestern Weinan. In terms of the modern MAP observations and geographic distribution pattern, the MAP values we obtained in Weinan are close to those obtained in Baoji. Indeed, our MAP reconstructions slightly exceed the MAP values reconstructed by ^10^Be in Baoji, spanning almost the whole 430 ka. Moreover, the MAP changes observed between the glacial and interglacial periods are nearly concentrated within similar ranges when the brGDGT-MAP model and ^10^Be are used. However, we revealed that opposite variations existed between the MAP values reconstructed by ^10^Be and MAPc values we reconstructed by using brGDGTs when the glacial and interglacial conditions were altered (Fig. 2, light-yellow bars). The ^10^Be-derived MAP is weakly correlated with the Chinese cave-derived δ^18^O (Fig. 2e) but aligns with global ice-volume variations (Fig. 2g, i)^41^, which may be affected by 65°N summer insolation in the Milankovitch hypothesis^42^. Thus, previous authors have argued that Chinese cave-derived δ^18^O is governed by low-latitude insolation rather than high-northern-latitude insolation (Fig. 2h).

Nevertheless, our reconstructed MAPc values corresponded well with the Chinese cave-derived δ^18^O, which is a significant proxy of EASM intensity and is also likely affected by precipitation, soil moisture and transportation paths^43^. Our MAPc results do not match the SWC and MAT variations that occurred in the last 430 ka (Fig. 3a). We found that 23-ka cycles dominated MAPc; these results do not correspond with the 100-ka cycles observed in SWC and MAT (Fig. 2a, c, f). A previous study also found a close and consistent relationship between MAT and SWC at glacial–interglacial scales^44,45^. In addition, we found that our MAPc results had relatively weak relationships with MAT and SWC (Fig. 3a). The reconstructed MAT and potential SWC (MBT’) values showed strong positive correlations with each other, and they were both negatively connected with soil pH. Concerning the co-vary relationship between MAT and SWC that might potentially be affected by the calibration model’s parameters we used, we have tested the different temperature (four models) and SWC (two models) calibration models, which show the same variation trends between all MAT and SWC reconstructions (Supplementary Fig. 17). These relationships are similar to the relationships between ^10^Be-MAP and the three paleoclimatic and paleoenvironmental factors we reconstructed (MAT, soil pH, and SWC) (Fig. 3b). As a result, the real atmospheric MAP might have a weak connection with the reconstructed MAT and SWC on glacial–interglacial temporal scales. Furthermore, combined with other studies and our results^45^, we concluded that the environmental SWC variations in the CLP are more affected by MAT than by MAP, due to the greater water evaporation and sparse vegetation cover in this area.

**Fig. 4:**
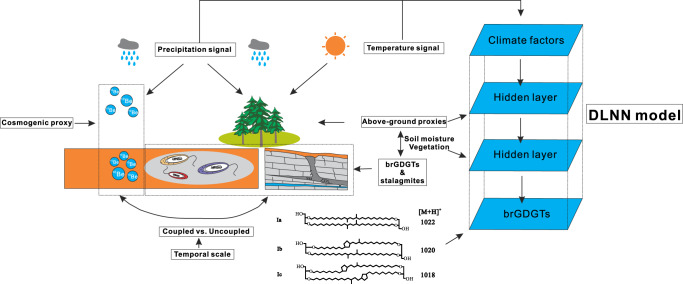
Schematic diagram reflecting the divergent responses between cosmogenic proxies and proxies of underground sources. All the elements are created and hand drawn by the first author of this article (Y.Z.).

The isotope ^10^Be, as a cosmogenic proxy, may more directly record atmospheric precipitation, while belowground proxies (i.e., brGDGTs and stalagmite δ^18^O) may be affected by surface variations^27,46^ (i.e., vegetation and SWC, Fig. 4). Compared with the promising SWC proxy, MBT’ (Fig. 2f), which can be applied in paleoclimate reconstructions when IR_6ME_ > 0.5 (Supplementary Fig. 16)^24,36^, we reveal that the uncoupled sections between ^10^Be-MAP and our results may be more strongly affected by SWC and MAT. ^10^Be-MAP is weakly connected with brGDGT-MAP when glacial and interglacial periods alternate, and this relationship becomes a positive connection when these periods are eliminated (Supplementary Fig. 18).

**Fig. 5:**
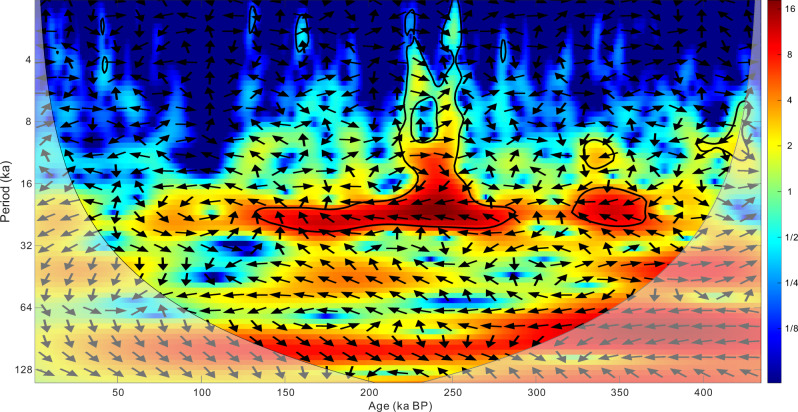
Cross wavelet spectral analysis between high-resolution Sanbao δ^18^O and mean annual average precipitation (MAP) we reconstructed in Weinan. Cross wavelet spectral output the same time resolution (1 ka) between the δ^18^O and MAP though linear interpolation. The arrows that pointed to the left indicate the reverse phase change of the two variables and the bigger value indicated by different colors represents the higher relationship of the two variables.

The brGDGTs and cave-derived δ^18^O may contain SWC and MAT signals and may only represent changes in the EASM intensity during some periods at glacial–interglacial scales. Other East Asian monsoon proxies, magnetic susceptibility and mean grain size, also show the same trends in these periods (Fig. 2b). In addition, we reconstructed relatively low soil pH values and warm environments in these periods (Fig. 2a), and the temperature variations in the Weinan profile obtained based on the phytoliths are shown to be similar to our results^18^. Previous studies have recorded that the climate in the continental interior had considerably diverse fluctuation forms in the glacial–interglacial cycle and even on shorter timescales^47^. Indeed, both SWC and temperature in monsoonal East Asia covaried with global ice volume and greenhouse gas (GHG) variations^45^. Combined with another vegetation-based biomarker, long-chain *n*-alkanes, which are derived from plant leaf waxes, can transport signals of changes in plant sources and past climate. The carbon preference index (CPI) and the average chain length (ACL) values revealed a relatively high SWC during the glacial and interglacial transition, we further confirmed these SWC fluctuations in long-term climate change (Supplementary Fig. 13). As a result, both brGDGTs and cave-derived δ^18^O may deviate from the real precipitation variations when MAP and SWC are decoupled; instead, they may denote reliable EASM proxies at glacial–interglacial scales. Indeed, the MAPc we reconstructed in Weinan shows the same primary cycle with the Sanbao cave-derived δ^18^O at the period of 23 ka. In contrast, during this primary period (i.e., 23 ka), they demonstrate an anti-phase variation trend (Fig. 5). Furthermore, brGDGTs have significant temporal and spatial superiority compared with stalagmite proxies, can be preserved in the environment for up to at least 55 Ma and do not have geographic restrictions^22^.

In summary, both brGDGTs and cave-derived δ^18^O may contain SWC and MAT signals and do not always maintain the same changes as those observed in MAP at glacial–interglacial scales. Although these proxies do not reflect rainfall changes at all times, they can represent long-term variations in EASM intensity. In addition, the utilization of our brGDGT-MAP model can vastly enrich EASM records in both temporally and spatially in the future.

## Methods

### Materials

Weinan city is located in the middle reaches of the Yellow River and in the southern part of the Loess Plateau (34°13’–35°52’N, 108°58’–110°35’E) (Fig. 1). It has a temperate semihumid, semiarid climate. The modern MAT observations indicate a value of 13.8 °C, and MAP is 570 mm; these values were obtained from the China meteorological data network, comprising the meteorological data of 2000–2015 (http://data.cma.cn/). Weinan has four distinct seasons, with hot and rainy conditions occurring in the same season. Much of the annual precipitation falls from June to August. The Weinan profile contains 42.8 m of loess–paleosol sequences (LPSs), including five paleosol layers from S_0_–S_4_ and five loess layers from L_1_–L_5_ and covering five glacial–interglacial cycles. The sampling method involved collecting one sample every 10 cm without interruption. A total of 427 samples were collected from this profile.

### Modern brGDGTs dataset and MAP dataset

Previously published brGDGTs data from surface soil samples were extracted using an established brGDGT-MAP model. The surface soil samples contain various types of soil and cover nearly all climatic and latitudinal zones. These datasets contain 712 surface soil samples, which all have separated 5-methyl and 6-methyl brGDGTs isomers (Table 1). To reduce the errors in collecting data from different laboratories, the MAP datasets we entered into the brGDGT-MAP model were all published in their previous studies, and we calculated the fractional abundances of each brGDGTs compound for each sample (Table 1), although there were no data regarding changes in soil occurring based on the brGDGTs indices among various laboratories. To eliminate and test the error of the previous MAP dataset, in this study, we also extracted each soil site’s multiyear MAP (1990–2020) through TerraClimate, which is a dataset of high-spatial-resolution monthly climate for global terrestrial surfaces (1/24°, ∼4 km)^48^. TerraClimate datasets reveal significant advances in the overall mean absolute error and enhance spatial realism compared with coarser resolution gridded datasets. Supplementary Fig. 3 shows that the two MAP datasets have high correlations, with only a few sites exhibiting large deviations. In this study, we entered these two MAP datasets into the DLNN model to obtain the most suitable DLNN-MAP model.

### Grain-size and magnetic susceptibility measurements

Samples at 10-cm intervals were dried in an oven at 40 °C for 3 days. Then, 0.2 g of each sample was weighed using a clean beaker with an electronic balance. Then, 10 ml of 30% H_2_O_2_ and 10 ml 10% HCl were added to remove organic matter and carbonate, respectively. Before the grain-size measurement, 0.05 mol/L (NaPO_3_)_6_ was added, and the solutions were placed in an ultrasonic machine for 10 min. The magnetic susceptibility of the samples were measured with an MS-2B Bartington meter. The grain-size was measured using a Mastersizer 2000 produced by Marvern Company in the UK, with an error of less than 1%.

### Chronology

We used the ages of LPS control points on the Loess Plateau to obtain the age of each sample in the Weinan profile^40^. We used the magnetic susceptibility as an indicator of the accumulation rate^39^ combined with the U–^230^Th-dated oxygen isotope records from Sanbao caves in central China^14^. Each sample’s magnetic susceptibility was analyzed at 10-cm intervals (Supplementary Fig. 7). The calculation was as follows:

1 $${T}_{{{{{{\rm{m}}}}}}}={T}_{1}+\frac{\left({\sum }_{i=1}^{m}{a}_{i}{s}_{i}\right)\left({T}_{2}-{T}_{1}\right)}{{\sum }_{i=1}^{n}{a}_{i}{s}_{i}}$$

where *T*_1_ and *T*_2_ indicate the ages of the control points, *a*_i_ indicates the thickness of the layer, and *s*_i_ indicates the magnetic susceptibility of the layer.

### GDGTs analysis

Lipids in a total of 238 LPS samples were extracted, including the 198 samples reported in ref. 49. Forty samples at depths from 34.9 m to 43.7 m were selected every 20 cm intervals from the Weinan profile, and dried in an oven at 40 °C for 3 days. Afterward, the loess and paleosol samples were ground into powder and passed through a 60-mesh sieve. Each sample was weighed and extracted with 80 ml of methanol: dichloromethane (DCM) (1:9, v/v) using accelerated solvent extractors (ASE 100 or 150, Dionex, USA). The temperature and pressure were set at 100 °C and 1400 psi, respectively. Then, the lipid extracts were condensed in a rotary evaporator at 40 °C and separated into apolar and polar fractions on a flash silica gel column (0.7 cm i.d. and 1.5 g activated silica gel) chromatography using *n*-hexane and methanol as eluents, respectively. All polar components were passed through a 0.45-µm PTFE syringe filter. All apolar and polar compositions were dried under a gentle stream of nitrogen gas.

The GDGTs were analyzed by using an Agilent 1200 series liquid chromatography-atmospheric pressure chemical ionization-6460A triple quadrupole mass spectrometry (LC-APCI-MS/MS). Ten microlitres of C_46_ GTGTs (0.001157 μg/μl) were added to each polar fraction, and the samples were then dissolved in 300 μl of *n*-hexane: iso-isopropanol (IPA) (98.2:1.8, v/v)). Two silica gel columns in series (150 mm × 2.1 mm, 1.9 μm, Thermo Finnigan; USA) were used for the separation of 5-methyl and 6-methyl brGDGTs, with the column temperature kept at 40 °C. The mass spectrometry settings were as follows: the vaporizer pressure 60 psi, the vaporizer temperature 400 °C, the flow rate of dry gas (N_2_) 6 l/min, drying gas temperature 200 °C, the capillary voltage 3500 V, the corona current 5 μA (∼3200 V), and a single-ion monitoring mode (SIM) was used^50^, targeting the protonated molecular ions ([M + H]^+^) 1304, 1302, 1300, 1298, 1296, 1292, 1050, 1048, 1046, 1036, 1034, 1032, 1020, 1018, and 744.

The MAT_mr_ proxy was calculated to identify the changes that occurred in the mean annual temperature in the Weinan section over the last 430 ka. The calculation was as follows^24,51^.

2 $${{MAT}}_{{mr}} 	=7.17+17.1*[{Ia}]+25.9*[{Ib}]+34.4*[{Ic}]-28.6*[{IIa}]\,(n=222,\,{R}^{2} \\ 	=0.68,\; {RM}{SE}=4.6 {\deg} {{{\rm{C}}}},\,P \; < \; 0.01)$$

3 $${{MAT}}_{{mr}}=5.58+17.91*[{Ia}]-18.77*[{IIa}]$$

4 $${MAT}({SSM})=	 20.9-13.4*[{IIa}+{IIa}^{{\prime}}]-17.2*[{IIIa}+{IIIa}^{{\prime}}]\\ 	 -17.5*[{IIb}+{IIb}^{{\prime}}]+11.2*[{Ib}]$$

5 $${MAAT}=0.81-5.67*{CBT}+31.0*{MBT}^{{\prime}}$$

The soil pH was calculated using the following formulas^24^.

6 $${pH}=7.15+1.59*{CBT}^{{\prime}}(n=221,\,{R}^{2}=0.85,\,{RMSE}=0.52,\, P \, < \,0.0001)$$

7 $${{CBT}}^{{\prime} }=-{{\log }}\frac{{Ic}+{II}{a}^{{\prime}}+{II}{b}^{{\prime}}+{{IIc}}^{{\prime} }+{{IIIa}}^{{\prime} }+{III}{b}^{{\prime} }+{{IIIc}}^{{\prime} }}{{Ia}+{Ib}+{Ic}}$$

SWC is well correlated with MBT’ when IR_6ME_ > 0.5, and these proxies were calculated using the following expressions:

8 $${{MBT}}^{{\prime} }=\frac{({Ia}+{Ib}+{Ic})}{({Ia}+{Ib}+{Ic}+{IIa}+{{IIa}}^{{\prime} }+{IIb}+{{IIb}}^{{\prime} }+{IIc}+{{IIc}}^{{\prime} }+{IIIa}+{IIIa}^{\prime} )}$$

9 $${{IR}}_{6{ME}}=\frac{\sum (C6-{methylated\; brGDGTs})}{\sum {brGDGTs}}$$

10 $${{MBT}^{\prime} }_{6{ME}}=\frac{({Ia}+{Ib}+{Ic})}{({Ia}+{Ib}+{Ic}+{{IIa}}^{{\prime} }+{{IIb}}^{{\prime} }+{{IIc}}^{{\prime} }+{IIIa}^{\prime} )}$$

where the Roman numerals indicate different brGDGTs structures (Supplementary Fig. 1).

### Principal component analysis (PCA)

CANOCO version 5 software was utilized to reveal the relationships among various environmental factors. The first PCA figure (Fig. 3a) was generated for the environmental factors MAT, MAPc, SWC, and pH. These variables are based on the same dataset (238 LSPs samples from Weinan profile) without any data transformation. The second PCA figure (Fig. 3b) was generated for the environmental factors MAT, MAP (based on ^10^Be), SWC and pH, which were all in the transition of the glacial–interglacial after 430 ka BP on the CLP. As the two LSPs profile had similar sedimentation rates, we obtained the same chronological control through linear interpolation in those transition periods. All datasets passed the Gaussian distribution test in this study.

### Cross wavelet analysis

Compared with wavelet special analysis, cross wavelet analysis is even more complicated. The wavelet cross-spectrum can be defined as follows:

11 $${CS}\left(b,\, a \right)={m}_{1c}(b,\, a){m}_{2c}(b,\, a)$$

where ${m}_{1c}$ and ${m}_{2c}$ describe the covarying fractions of the overall spectra given by:

12 $${m}_{1}\left(b,\, a \right)={m}_{1c}\left(b,\, a \right)+{m}_{1i}(b,\, a)$$

13 $${m}_{2}\left(b,\, a \right)={m}_{2c}\left(b,\, a \right)+{m}_{2i}\left(b,\, a \right)$$

where ${m}_{1i}$ and ${m}_{2i}$ are independent contributions to the variance.

Overall, this is a multipart function that may be decomposed into amplitude and phase:

14 $${CS}\left(b,\, a \right)={{{{{\rm{|}}}}}}{CS}\left(b,\, a \right){{{{{\rm{|}}}}}}{{\exp }}(i\;{{\arg }}({CS}(b,\, a)))$$

In this study, a and b represent the array of reconstructed MAPc and the Sanbao speleothem δ^18^O, respectively.

### Multiple regression linear model

To compare the precision of the DLNN-MAP model we established, we set up a multiple regression linear model based on all 6-methyl brGDGTs except Ib. The basis of the model is defined as:

15 $$y=a+{b}_{1}{x}_{1}+{b}_{2}{x}_{2}\ldots+{b}_{n}{x}_{n}$$

where *y* represents MAP, *x* represents all 6-methyl brGDGTs and Ia and Ic, and a, *b1, b2…bn* represent the partial regression coefficients. *n* represents the number of 6-methyl we entered into the model (in this study, *n* = 8).

The multiple correlation coefficient (R) was defined as follows:

16 $$R=\sqrt{\frac{{\sum }_{i=1}^{n}{({\hat{y}}_{i}-\bar{y})}^{2}}{{\sum }_{i=1}^{n}{({y}_{i}-\bar{y})}^{2}}}$$

where ${y}_{i}$ represents the actual observed value, ${\hat{y}}_{i}$ represents the calculation value and $\bar{y}$ represents the mean value of all observed data.

The *t* statistic is used to test the validity of regression coefficients, and it can be defined as follows:

17 $${t}_{{b}_{j}}=\frac{{b}_{j}}{{s}_{{b}_{j}}}$$

18 $${s}_{{b}_{j}}=\sqrt{{p}_{{jj}}}*s$$

19 $$s=\sqrt{\frac{1}{n-m-1}\mathop{\sum }\limits_{i=1}^{n}{({y}_{i}-{\hat{y}}_{i})}^{2}}$$

20 $$P={({p}_{{jj}})}^{-1}=\mathop{\sum }\limits_{i=1}^{n}({x}_{{ij}}-{\bar{x}}_{j})({x}_{{ik}}-{\bar{x}}_{k})$$

where ${b}_{j}$ represents the regression coefficient of ${x}_{j}$, *n* represents the number of samples and *m* represents the number of variables.

The *F* statistic is used to test the linear relationship of variables and can be defined as follows:

21 $$F=\frac{1}{m{s}^{2}}\mathop{\sum }\limits_{i=1}^{n}{({\hat{y}}_{i}-\bar{y})}^{2}$$

The variance inflation factor (VIF) is used to measure collinearity between variables and can be defined as follows:

22 $${{VIF}}_{j}=\frac{1}{1-{R}_{j}^{2}}$$

As shown in Supplementary Fig. 5, we found no obvious collinearity between different variables. However, there are fewer contributions in IIc’, IIIa’, IIIb’, and IIIc’ in the multiple regression linear model we established, and the value of *t* cannot attain the 95% confidence level (Table 2). The results of both the training dataset and extrapolated experimental dataset (Supplementary Fig. 6), although they seem good (*R*^2^ = 0.44 and 0.46, respectively), still have a considerable gap compared with the DLNN-MAP model. Especially when MAP > 1500 mm, the multiple linear model is unable to forecast the real MAP. These results all indicate that the MAP influence on the brGDGTs compounds is not a simple linear relationship; instead, we suggest that there are complex pilot processes between them.

**Table 2:** List of the parameters of the multiple linear model

Model	Coefficient	*t*	Significance	VIF
a	1428.527	41.153	0.000	
b_1_	0.116	0.526	0.599	2.561
b_2_	1524.901	2.641	0.009	2.638
b_3_	−2070.924	−7.983	0.000	2.792
b_4_	−941.825	−1.190	0.235	2.365
b_5_	4507.380	0.432	0.666	2.191
b_6_	−1158.136	−3.672	0.000	2.347
b_7_	−3786.251	−5.061	0.000	1.061
b_8_	11643.395	−0.421	0.674	1.305

### DLNN models

DLNNs usually contain an input layer, a few hidden layers, and an output layer. A conceptual structure of the DLNN model was established for forecasting MAP values. The first layer accepts input signals that are various combinations of brGDGTs. The relationships among different variables are processed and analyzed in the hidden layers. The final class output is presented in the output layer; in this study, the output is the MAP reconstruction at the study site.

The rectified linear unit (ReLU) activation function is applied in each neuron of the hidden layer, which is computationally simpler than the traditionally applied sigmoid function. The function of the ReLU activation function is given as follows:

23 $$f\left(x\right)=\left\{\begin{array}{c}x,\, x \, > \, 0 \\ 0,\, x \, \le \, 0\end{array}\right.={{{{{\rm{max }}}}}}(0,\, x)$$

where *x* represents an input signal to a neuron and *f* represents the activation function.

Furthermore, the bias between the measured and forecasted output values is reflected by the loss function. The loss function applied herein is the MAE (mean absolute error), which is given as follows:

24 $${MAE}=\frac{1}{N}\mathop{\sum }\limits_{i=1}^{n}{{{{{\rm{|}}}}}}T-Y{{{{{\rm{|}}}}}}$$

where *N* is the number of training data points, and *T* and *Y* represent the measured output value and the forecasted class value, respectively.

To realize the backpropagation framework, the derivative of the ReLU activation function needs to be acquired. According to the definition of the ReLU, the derivative is shown as follows.

25 $$f{^\prime} \left(x\right)=\left\{\begin{array}{c}1,\; x \, > \, 0 \\ 0,\; x \, \le \, 0\end{array}\right.$$

Given a minibatch of m training samples obtained from the training set {x(1), x(2)…, x(m)} and their corresponding targets T(*i*) (*i* = 1,2…, m), the gradient used in the backpropagation framework is shown as follows:

26 $$f=\frac{1}{m}\mathop{\sum }\limits_{i=1}^{n}\frac{\partial L}{\partial w}$$

where *L* is the loss function; *w* represents the network weights; and *n* = 1 is the number of output values (MAP).

In addition, considering that the adaptive moment estimation algorithm (Adam) was proven to be an effective neural network training method with a fast convergence speed and great classification performance, we applied this algorithm to train the DLNN model for MAP forecasting in this study. Adam has two biased equations, which are shown as follows:

27 $$a={\rho }_{1}a+(1-{\rho }_{1})g$$

28 $$b={\rho }_{2}b+(1-{\rho }_{2})g\times g$$

where ${\rho }_{1}=0.9$ and ${\rho }_{2}=0.999$ are exponential decay rates; *g* is the gradient; and $\times$ represents an elementwise product operator.

After this calculation, the correct biases in the above two moments are given as follows:

29 $${a}_{c}=\frac{a}{1-{\rho }_{1}^{t}}$$

30 $${b}_{c}=\frac{b}{1-{\rho }_{2}^{t}}$$

where *t* represents the current time step.

Moreover, the update of the network weights is shown as follows:

31 $${\triangle }_{w}=-\lambda \frac{{a}_{c}}{\sqrt{{b}_{c}}+\epsilon }$$

where $\lambda=0.001$ represents the learning rates and $\epsilon={10}^{-8}$ is a constant used to ensure numerical stability.

Eventually, the DLNN parameters can be updated according to the following formula.

32 $$w \,=\, w \,+\, {\triangle }_{w}$$

### brGDGT-MAP models

We entered 9 brGDGTs compounds (all 6-methyl brGDGTs; each compound entered in the model is the percentage of all brGDGTs in the surface soil) into the input layer of the DLNN; these compounds are closely related to soil moisture. Then, we selected 533 surface soil samples as the training dataset and 179 surface soil samples as the validation dataset, both of which satisfied the principle of randomness. We assessed the precision of the model using forecast data *R*^2^ and root mean square error (RMSE) values.

Through several parameters applied in the DLNN model, we found that the frequency of training and the number of neurons play the most significant roles in the brGDGT-MAP models. In addition, four hidden layers containing the other DLNN parameters allow the model to become more stable (detailed parameters are shown in Supplementary Fig. 7). To test the best frequency of training and the number of neurons in each hidden layer, we set a series of gradients to test the model to find the most suitable combination. As shown in Supplementary Fig. 8, for the frequency of training, we set the minimum and maximum training times to 1000 and 1500, respectively, with 100 times as the interval. We also set the numbers of neurons from 160 to 260 with a 20-neuron interval.

Testing the weights of different compounds in the DLNN model and determining whether it was essential to eliminate some compounds that may make the dataset redundant were also required. Based on the model in which the Ib parameter was removed, we also set a series of experiments to test the effects of the different 6-methyl isomers on the predicted MAP values. Then, we made seven attempts to test the forecast effect of the brGDGT-MAP models by removing the Ic, IIa’, IIb’, IIc’, IIIa’, IIIb’, and IIIc’ parameters (Supplementary Fig. 9). Then, we obtained the best brGDGT-MAP model (Supplementary Fig. 10).

### Comparison of various ANN structures

To improve the accuracy of our brGDGT-MAP models and the models’ universality, we also tested more complex ANN structures and then compared them with our DLNN models.

#### RNN

A recurrent neuron network (RNN) is an artificial neural network in which nodes are directionally connected into loops. The essential feature of RNN is that there are both internal feedback connections and feedforward connections between processing units. The inner structure of RNN is similar to that of the human brain, which can learn to transform a lifetime of sensory input streams into an efficient sequence of motor outputs (Supplementary Fig. 11a). Therefore, the basis of the RNN is defined as follows:

33 $${h}_{t}=f\left(U \,*\, {X}_{t}+W \,*\, {h}_{t-1}\right)$$

34 $${o}_{t}={softmax}(V \,{h}_{t})$$

where X_t_ represents the input value at time *t*; *o*_*t*_ represents the output value at time *t*; *h*_*t*_ represents the memory value at time *t*; and *U*, *V*, and *W* are the parameters of this network. For the motivative function, we chose softmax.

#### LSTM

Long short-term memory networks (LSTM) are a special type of RNN that can learn long-term dependence and contain three gates (forget gate, input gate and output gate) and one memory cell. The horizontal line above the box is called the cell state, and it acts as a conveyor belt to control the flow of information to the next moment (Supplementary Fig. 11b). Therefore, the basis of LSTM is defined as follows:

35 $${C}_{t}={f}_{t}*{C}_{t-1}+{i}_{t}*{\widetilde{C}}_{t}$$

where ${C}_{t-1}$ represents the knowledge state of the model at time *t* − 1 and ${\widetilde{C}}_{t}$ represents the newly acquired information after entering new observations. ${f}_{t}$ and ${i}_{t}$ represent the weight parameters of ${C}_{t-1}$ and ${\widetilde{C}}_{t}$, respectively.

36 $${f}_{t}=\sigma ({W}_{f}\cdot \left[{h}_{t-1},\, {x}_{t}\right]+{b}_{f})$$

37 $${i}_{t}=\sigma ({W}_{f}\cdot \left[{h}_{t-1},\, {x}_{t}\right]+{b}_{i})$$

38 $$\kern0.9pc {\widetilde{C}}_{t}={{\tanh }}({W}_{c}\cdot \left[{h}_{t-1},\, {x}_{t}\right]+{b}_{c})$$

where ${h}_{t-1}$ represents the output value at time *t* − 1 and ${x}_{t}$ represents the new input value at time *t*. ${W}_{f}$ represents the motivative function in this study. We used tanh as the motivative function when our model was learning. Each new input may not have a positive impact on the machine, but it may also have a negative impact., ${b}_{f}$, ${b}_{i}$ and ${b}_{c}$ represent the random disturbances (white noise).

#### GRU

As mentioned above, the LSTM is proposed to overcome RNN’s inability to address remote dependence and the gate recurrent unit (GRU), a variant of the LSTM, keeps the effect of the LSTM while making the structure simpler.

Compared with the LSTM, the GRU only has two gates (update (*z*_*t*_) and reset (*r*_*t*_) gates). The update gate is used to control the degree to which the state information at the previous moment is brought into the current state. The larger the value of the update gate is, the more state information at the previous moment is brought in. The reset gate is used to control the degree to which the state information at the previous moment is ignored (Supplementary Fig. 11c). Therefore, the basis of the LSTM is defined as follows:

39 $${r}_{t}=\sigma ({W}_{r}\cdot [{h}_{t-1},\, {x}_{t}])$$

40 $${z}_{t}=\sigma ({W}_{z}\cdot [{h}_{t-1},\, {x}_{t}])$$

41 $${\widetilde{h}}_{t}={\tanh }({W}_{\widetilde{h}}\cdot [{{r}_{t}*h}_{t-1},\, {x}_{t}])$$

42 $${h}_{t}=\left(1-{z}_{t}\right)*{{r}_{t}*h}_{t-1}+{z}_{t}*{\widetilde{h}}_{t}$$

43 $${y}_{t}=\sigma ({W}_{o}\cdot {h}_{t})$$

where [] represents the connection of two vectors and * represents the multiplication of matrix elements. The *x*_*t*_ and *y*_*t*_ represent the input and output values at time *t*, respectively.

It can be seen from the above formula that the parameters to be learned are the weight parameters of *W*_*r*_, *W*_*z*_*, W*_*h*_, and *W*_*o*_. The first three weights are spliced; therefore, they need to be segmented during learning. These can be defined as follows:

44 $${W}_{r}={W}_{{rx}}+{W}_{{rh}}$$

45 $${W}_{z}={W}_{{zx}}+{W}_{{zh}}$$

46 $${W}_{\widetilde{h}}={W}_{\widetilde{h}x}+{W}_{\widetilde{h}h}$$

As we can find in the RNN, LSTM, and GRU models we reconstructed (Supplementary Fig. 12), the training datasets all show extraordinarily high *R*^2^ values (0.99, 0.99, and 0.99, respectively) and low RMSE values (0.36, 0.23, and 0.16, respectively). However, the validation datasets do not show good prediction ability compared with the DLNN. These results indicate that the two ANN structures are not suitable for MAP prediction based on brGDGTs, although their inner structures are more complex than those of the DLNN. The reason we suggested is that the RNN, LSTM and GRU are more appropriate to the massive amounts of data and the data that have obvious spatiotemporal characteristics. The great prediction precision in the training dataset and the poor performance in the extrapolated datasets indicate that the models based on the RNN, LSTM and GRU have significant overfitting. As a result, compared with other ANN structures, we concluded that our DLNN model is the most suitable one to forecast MAP based on brGDGTs.

### Environmental indicators of *n*-alkanes proxies

Long-chain *n*-alkanes in plant leaf waxes are universal in terrestrial environments and can deliver signals of variations in plant sources and past climate. They are widely distributed in surface soils and Quaternary sediments, especially in LPSs. In this study, due to the insufficient samples in Weinan profile, we only analyzed *n*-alkanes components for 40 LPS samples, which contain ages between 340 and 430 ka BP.

### Instrumental measurements

For the apolar fractions, a total of 40 samples in this study, mainly containing *n*-alkanes, were all investigated utilizing a Shimadzu 2010 gas chromatograph (GC) equipped with a flame ionization detector (FID) and a DB-5 fused silica capillary column (60 m $\times$ 0.32 mm $\times$ 0.25 μm film thickness) with helium as the carrier gas. The temperature of the GC oven was enhanced from 70 to 300 °C at a rate of 3 °C/min. Then, this temperature (300 °C) was maintained for 30 min. Finally, the concentrations of the *n*-alkane homologs were evaluated by assessing the peak area of the *n*-alkanes to that of the internal standard (cholane).

### Long-term paleoclimatic change

The carbon preference index (CPI) evaluates the relative abundances of odd vs. even-numbered *n*-alkanes. The CPI increases as the environmental aridity increases. The CPI indicated warm–wet periods and cold-dry periods in paleoclimate and corresponded well with the loess–paleosol cycle^52^. The average chain length (ACL) value is the weighted average of the different carbon chain lengths. The lower ACL value corresponds to the lower temperature in the research of LPSs. The variations in the ACL value have good coordination with the magnetic susceptibility and particle size. The *n*-alkane CPI^53^ and ACL^54^ are calculated as follows:

47 $${CPI}(1)=\frac{({C}_{23}+{C}_{25}+{C}_{27}+{C}_{29}+{C}_{31})+({C}_{25}+{C}_{27}+{C}_{29}+{C}_{31}+{C}_{33})}{2({C}_{24}+{C}_{26}+{C}_{28}+{C}_{30}+{C}_{32})}$$

48 $${CPI}\left(2\right)=\frac{1}{2}\left(\frac{{C}_{25}+{C}_{27}+{C}_{29}+{C}_{31}+{C}_{33}}{{C}_{24}+{C}_{26}+{C}_{28}+{C}_{30}+{C}_{32}}+\frac{{C}_{25}+{C}_{27}+{C}_{29}+{C}_{31}+{C}_{33}}{{C}_{26}+{C}_{28}+{C}_{30}+{C}_{32}+{C}_{34}}\right)$$

49 $${ACL}=\frac{{23C}_{23}+{25C}_{25}+{27C}_{27}+{29C}_{29}+31{C}_{31}+{33C}_{33}}{{C}_{23}+{C}_{25}+{C}_{27}+{C}_{29}+{C}_{31}{+C}_{33}}$$

Figure 13 shows the variations in CPI (Supplementary Fig. 13a) and ACL (Supplementary Fig. 13b) values in the Weinan profile from 340 to 430 ka BP. Compared with the MAP (Supplementary Fig. 13c) and SWC (Fig. 2e) reconstructions based on brGDGTs, we found that they all had a peak at ∼350 ka BP, which indicates relatively high soil moisture at approximately 350 ka BP.

### MAP reconstruction in the XRD section

In this section, we test the brGDGT-MAP model in the Xiangride (XRD) profile, which is located in the margin region of the East Asian monsoon (Fig. 1). With robust chronological control, we reconstructed the rainfall changes in 7000 years BP (Supplementary Fig. 14b). We found that MAP was ∼200 mm in the late Holocene, which approaches multiple modern observations in this region (180 mm). Moreover, we suggest that this region experienced the most humid period in the mid-Holocene, when the rainfall reached 600 mm. Afterward, the precipitation declined from 6000 to 4000 years BP and then increased and reached a peak value at ∼3000 years BP. Then, it had a drought trend until modern times.

We discovered that our brGDGT-MAP model could precisely capture rainfall dynamics based on the Weinan profile (Supplementary Fig. 14a) and XRD profile (Supplementary Fig. 14b). Combined with the most acceptable rainfall records in the Holocene (i.e., ^10^Be (Supplementary Fig. 14c), pollen in Gonghai (Fig. 1 and Supplementary Fig. 14d), and Dongge cave δ^18^O (Fig. 1 and Supplementary Fig. 14e)), we found the same precipitation peak values in the early Holocene and mid-Holocene. In addition, they all revealed a drought trend throughout the whole Holocene. We suggest that brGDGTs can become a robust proxy to reconstruct precipitation in the Holocene.

## Supplementary information

Supplementary Information

## Data Availability

The relevant data of Weinan LPSs profile are provided in the Supplementary Information Data file. Source data are provided with this paper.

## Source data

Source Data

## Code availability

The code used in this study is available from the corresponding author upon request.
