# Supplementary material for: Decoupled Asian monsoon intensity and precipitation during glacial-interglacial transitions on the Chinese Loess Plateau
Source: Nat Commun. 2022 Sep 14;13:5397. doi: 10.1038/s41467-022-33105-2 (PMC9474459; doi:10.1038/s41467-022-33105-2)
Supplement: Supplementary file 1 — Supplementary Information [file 41467_2022_33105_MOESM1_ESM.pdf]

Supplementary Information

This PDF file includes:

Supplementary Figure 1. Molecular structures of the brGDGTs.
Supplementary Figure 2. World map representing the locations of the surface soils used in this study (n=712).
Supplementary Figure 3. Linear relationship between the MAP we collected from previous study and the MAP_1 we extracted from TerraClimate.
Supplementary Figure 4. Linear relationships between the various brGDGTs isomers and MAP.
Supplementary Figure 5. The Pearson correlation coefficient between the MAP and various brGDGTs isomers in training brGDGTs datasets (n=533).
Supplementary Figure 6. The MAP values based on multiple linear regression model against the measured MAP values.
Supplementary Figure 7. Configuration of the DLNN model used for the MAP reconstructions based on various combinations of 6-methyl isomers.
Supplementary Figure 8. Heat maps of the different DLNN parameters.
Supplementary Figure 9. Test of brGDGT-MAP models.
Supplementary Figure 10. The MAP values obtained based on all 6-methyl isomers except Ib plotted against the measured MAP values.
Supplementary Figure 11. Configuration of the RNN, LSTM and GRU models
Supplementary Figure 12. The MAP predicted results of RNN, LSTM and GRU models.
Supplementary Figure 13. Variations in MAPc we reconstructed based on brGDGTs compared with n-alkanes proxies from 340 ka BP to 430 ka BP.
Supplementary Figure 14. The variations of MAP we reconstructed in Weinan and XRD compared with other precipitation results in EASM regions.
Supplementary Figure 15. Age-depth picks from the Weinan profile based on 427 samples.
Supplementary Figure 16. Variations in the IR_{6ME} values measured in the Weinan profile over the last approximately 430 ka BP.
Supplementary Figure 17. The comparison of different temperature and SWC calibration models in the Weinan profile based on brGDGTs.
Supplementary Figure 18. The linear relationship between brGDGT-MAP and ^{10}Be-MAP.

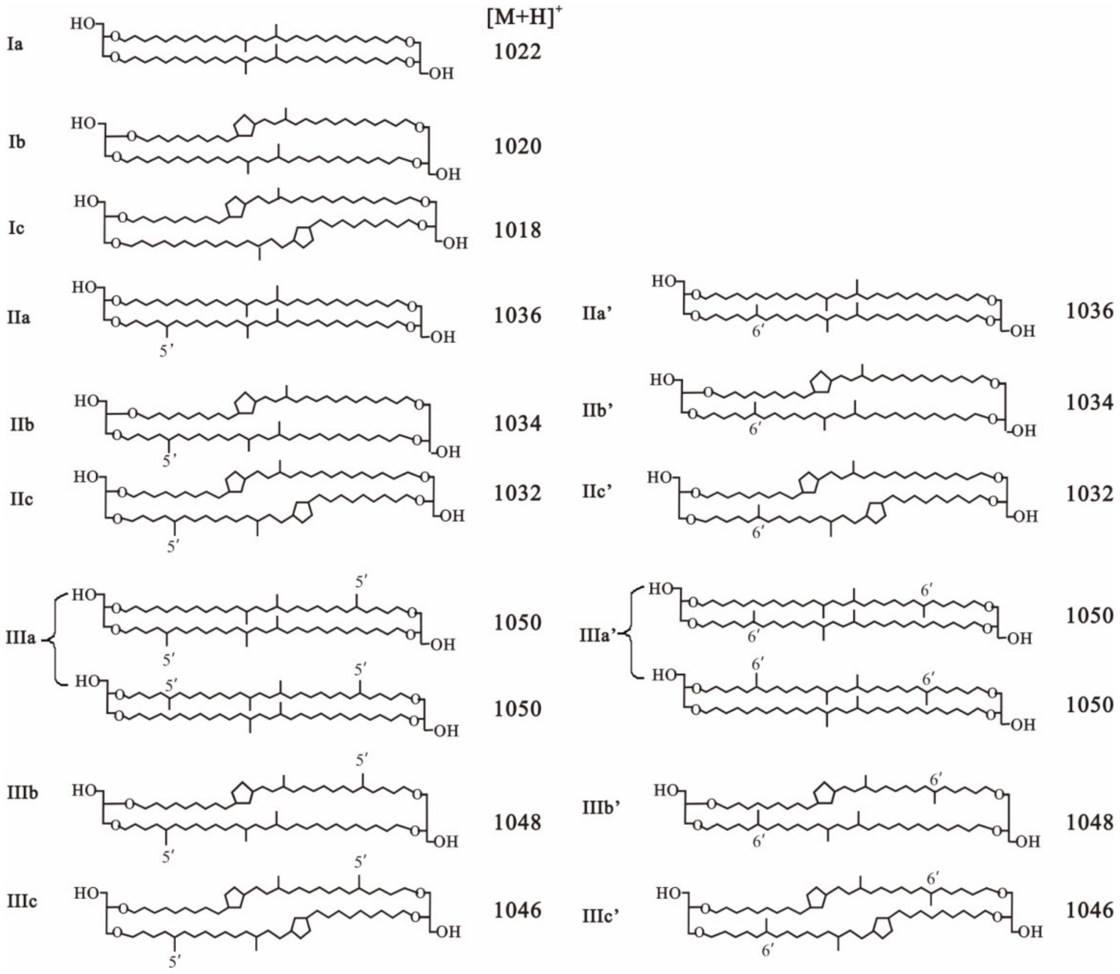

Supplementary Figure 1. Molecular structures of the brGDGTs. The Roman numerals indicate different brGDGTs structures

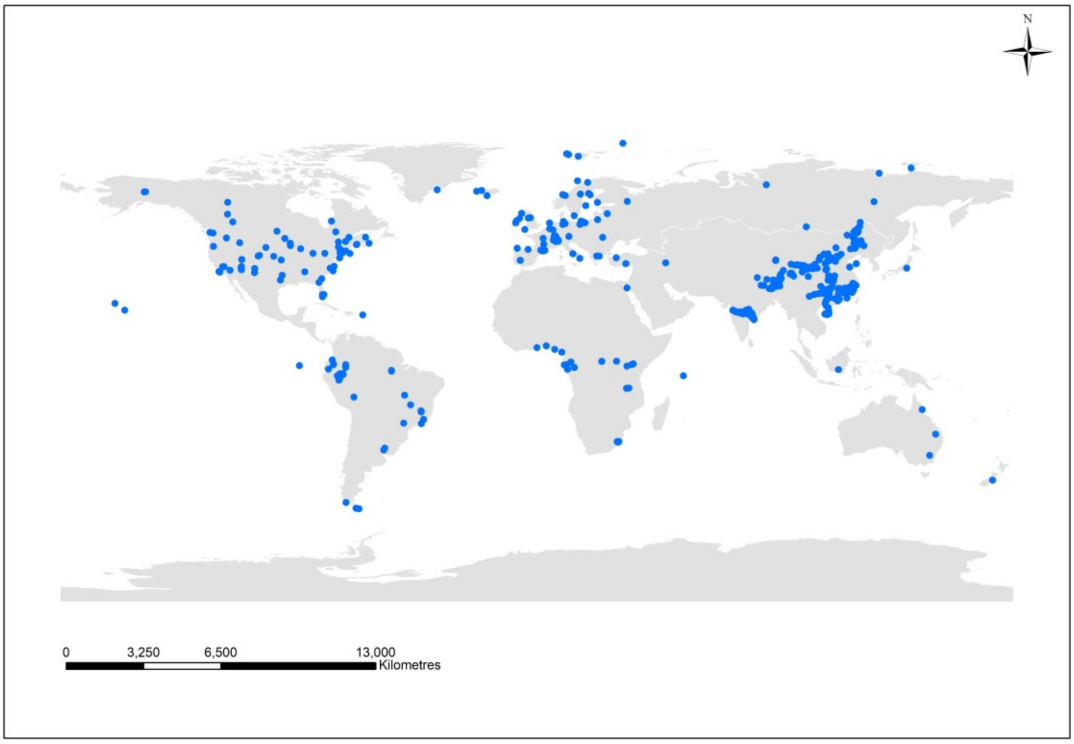

Supplementary Figure 2. World map representing the locations of the surface soils used in this study (n=712). The base map was generated using ArcGIS software.

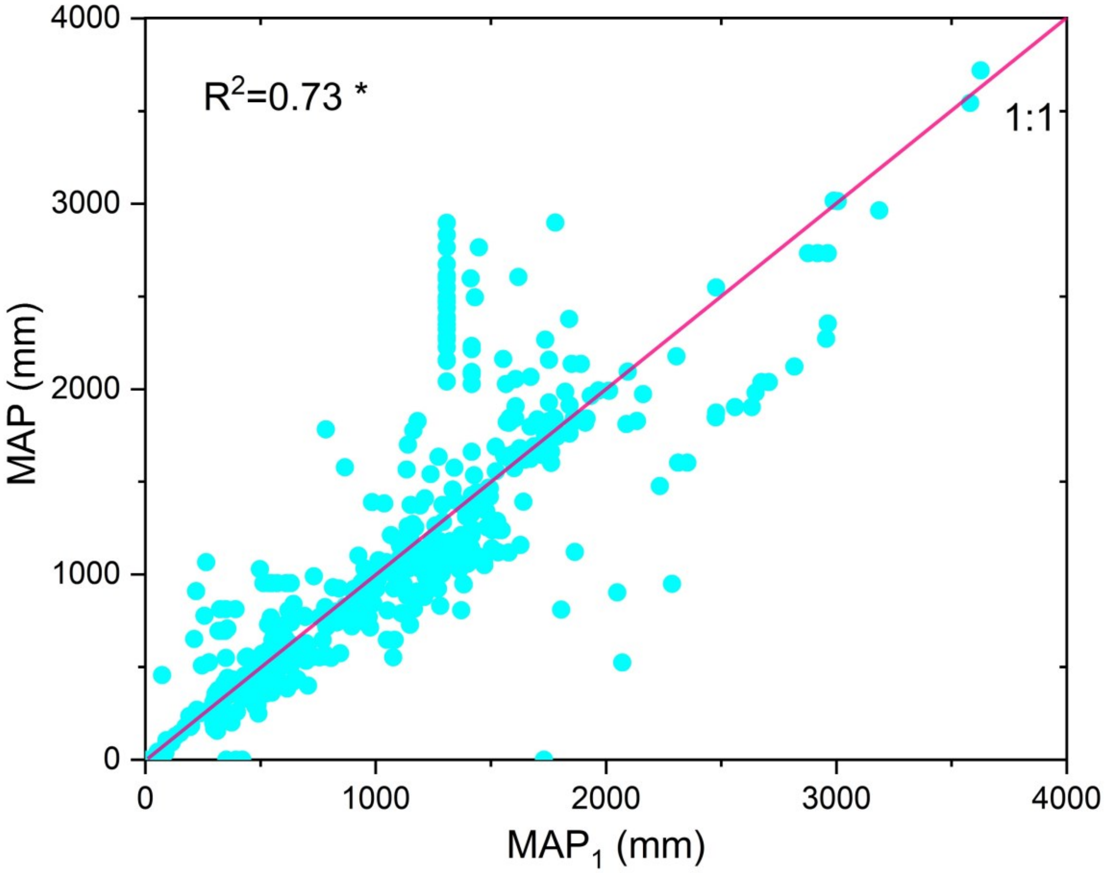

Supplementary Figure 3. Linear relationship between the MAP we collected from previous study and the MAP_1 we extracted from TerraClimate. * indicates the 99% confidence level.

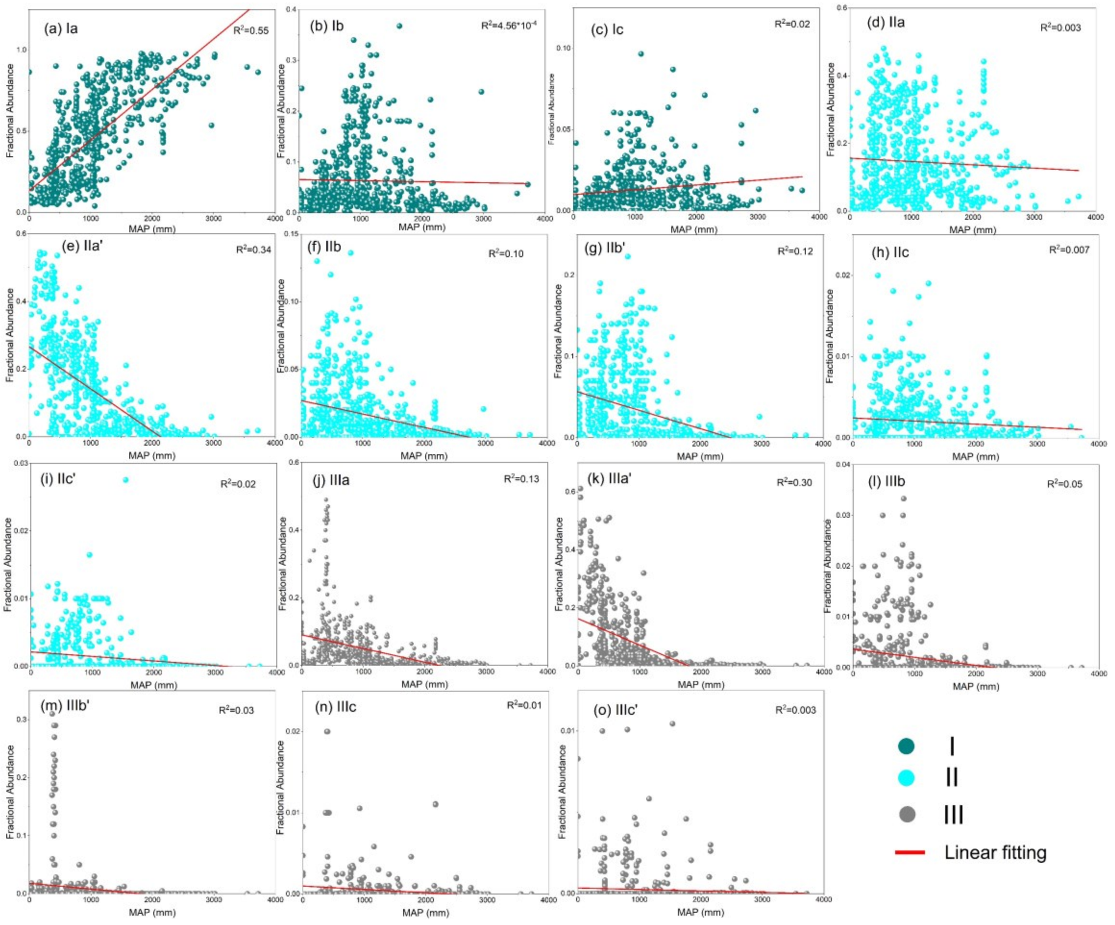

Supplementary Figure 4. Linear relationships between the various brGDGTs isomers and MAP. The green cycles represent series I brGDGTs, the blue cycles represent series II brGDGTs and the gray cycles represent series III brGDGTs. The red solid lines represent the line-fitting curves (n=712).

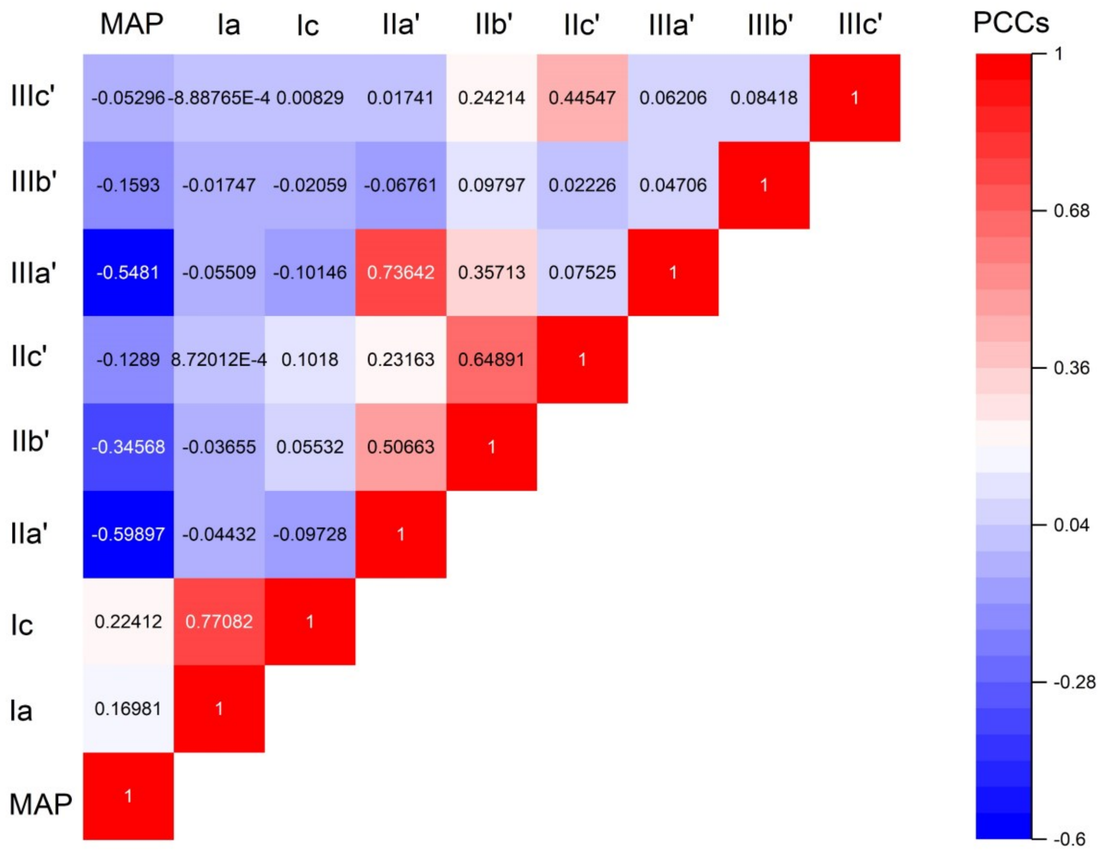

Supplementary Figure 5. The Pearson correlation coefficient between the MAP and various brGDGTs isomers in training brGDGTs datasets (n=533). Darker red squares represent higher positive correlation and darker blue squares represent higher negative correlation.

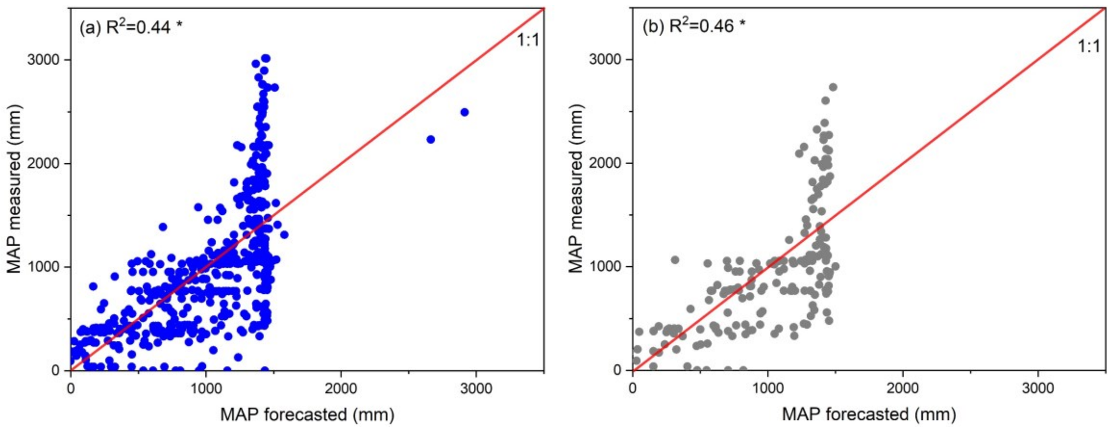

Supplementary Figure 6. The MAP values based on multiple linear regression model against the measured MAP values. (a) The training dataset of MAP (n=533) based on multiple linear regression model and (b) the validation dataset of MAP based on multiple linear regression model (n=179). * indicates the 99% confidence level.

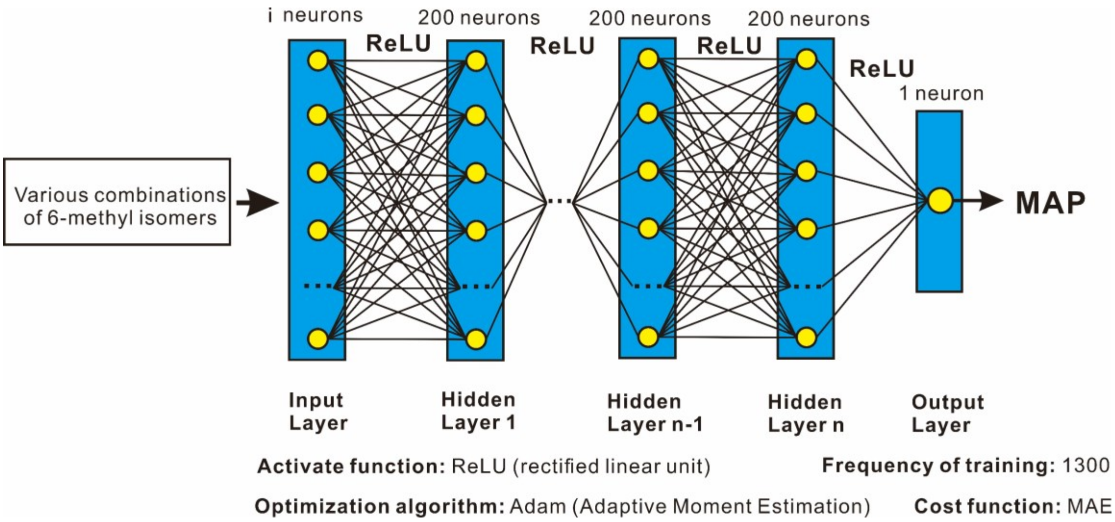

Supplementary Figure 7. Configuration of the DLNN model used for the MAP reconstructions based on various combinations of 6-methyl isomers. The term n represents the number of hidden layers in each model (in this study, n=4), and the term i represents the number of variables in the input layer.

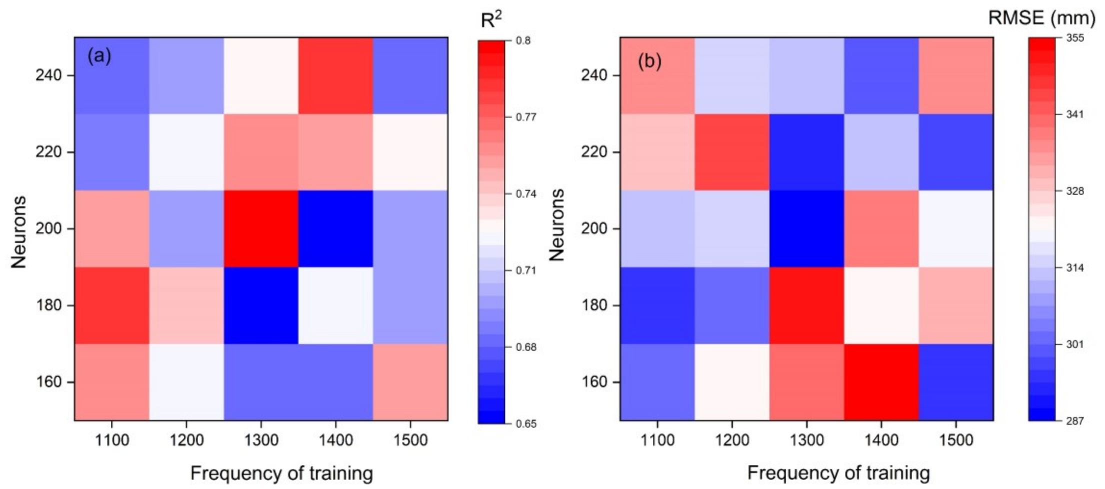

Supplementary Figure 8. Heat maps of the different DLNN parameters (frequency of training and numbers of neurons) corresponding to (a) R^2 and (b) RMSE (mm) values. (a) Darker red squares represent higher R^2 values. (b) Darker blue squares represent smaller RMSE values.

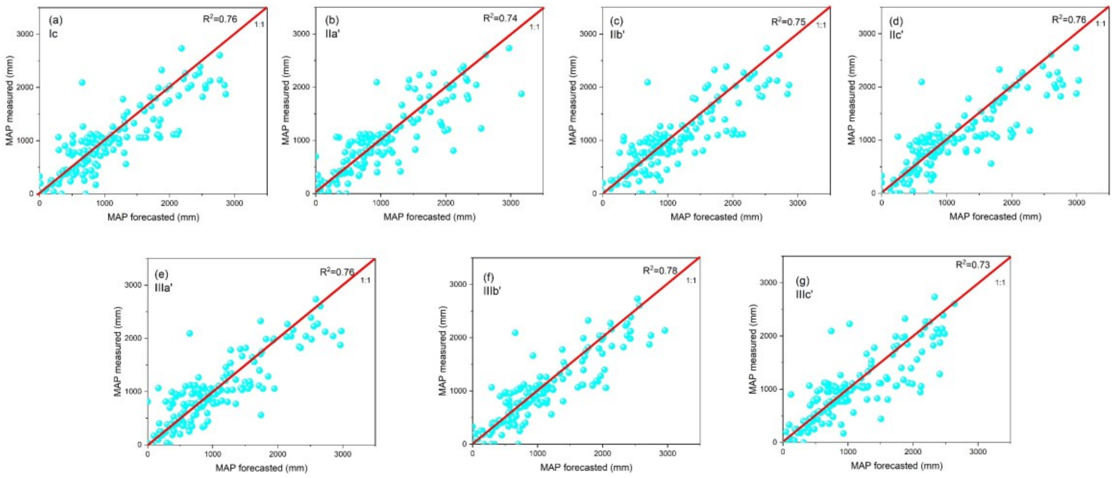

Supplementary Figure 9. From left to right and from top to bottom, the validation (n=179) brGDGT-MAP values: the MAP values obtained based on brGDGTs isomers (8 compounds, all 6-methyl isomers except Ib) in addition to Ic, IIa', IIb', IIc', IIIa', IIIb', and IIIc', plotted against measured MAP values.

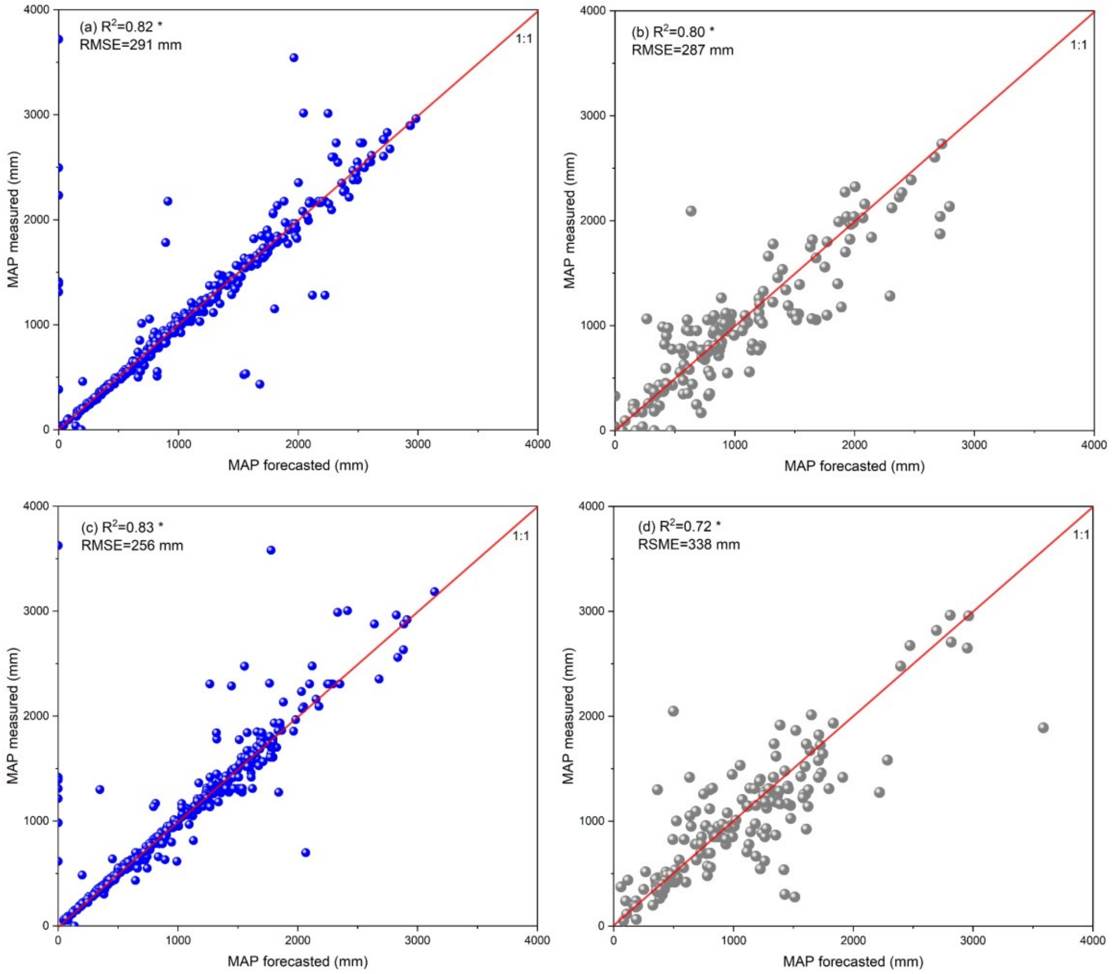

Supplementary Figure 10. The MAP values obtained based on all 6-methyl isomers except Ib plotted against the measured MAP values. (a) and (b), the measured MAP values we used are all collected from previous published studies. (c) and (d), the measured MAP values we used are extracted based on TerraClimate (multi-years MAP (1990-2020)). (a, c) The training dataset of brGDGT-MAP (n=533) and (b, d) the validation dataset of brGDGT-MAP (n=179). * indicates the 99% confidence level.

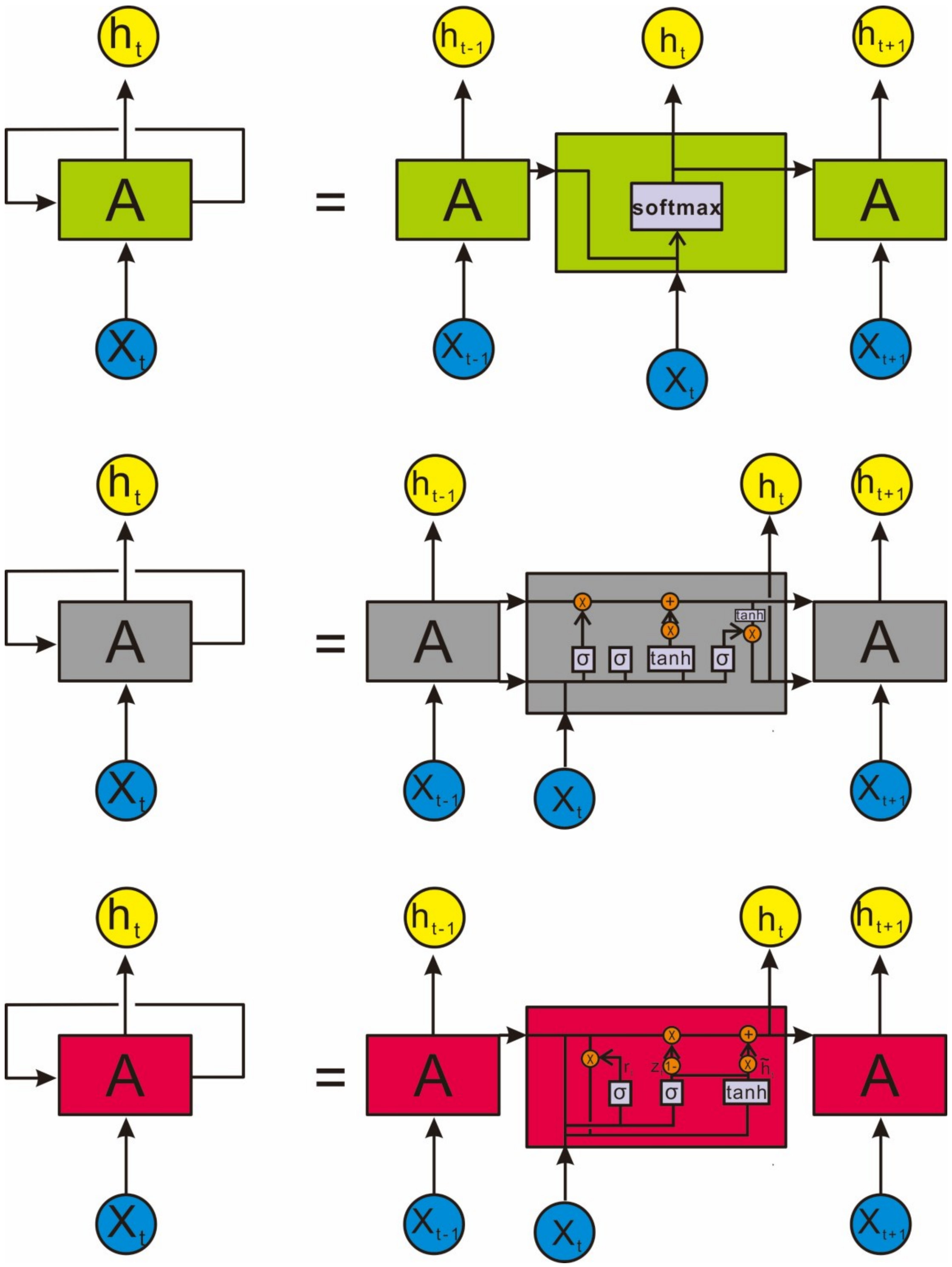

Supplementary Figure 11. Configuration of the RNN, LSTM and GRU model used for the MAP (h_t) reconstructions based on combinations of 6-methyl isomers (X_t). The t represents the orders of the input factors.

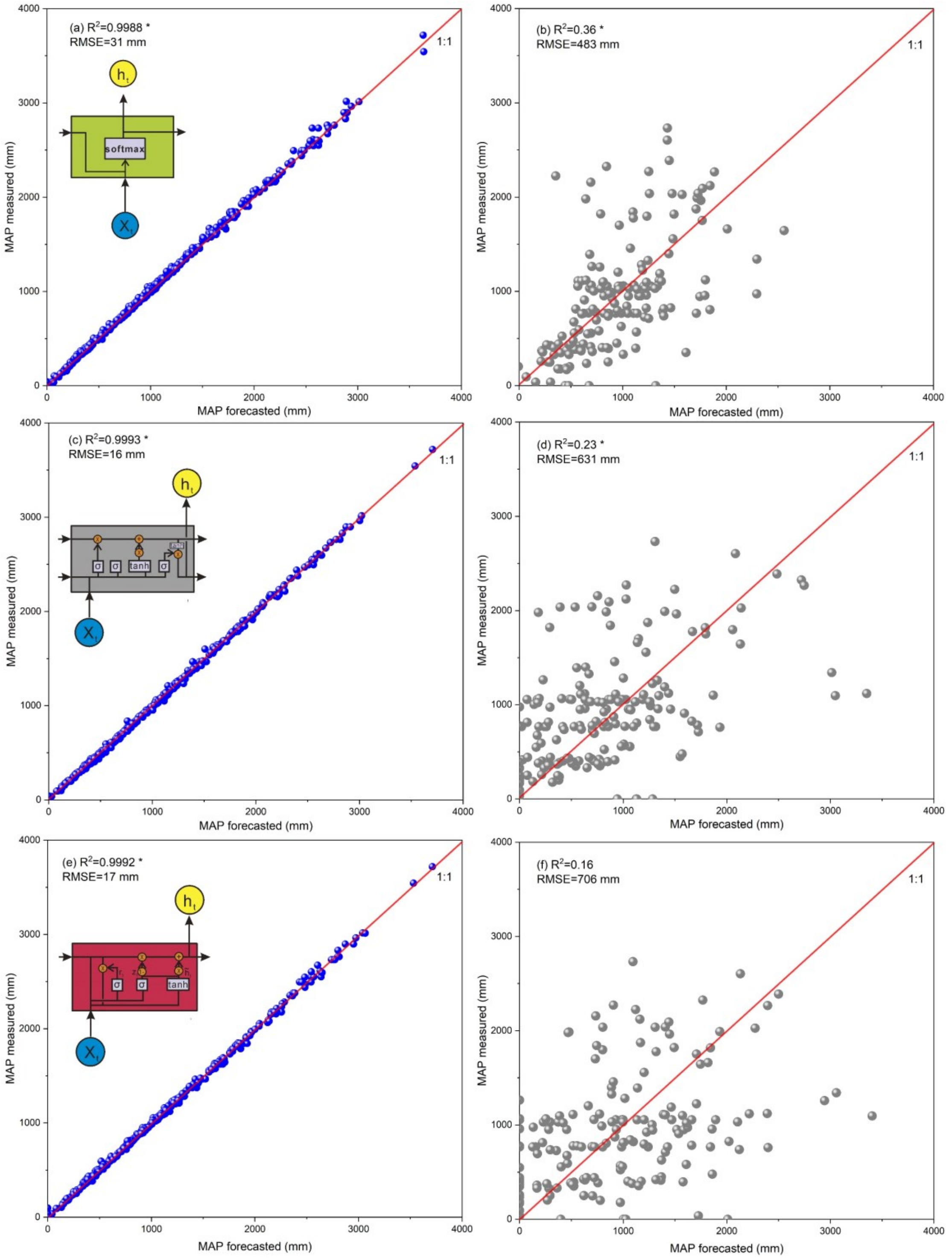

Supplementary Figure 12. The MAP values obtained based on all 6-methyl isomers except Ib plotted against the measured MAP values. For RNN model, (a), The training dataset of brGDGT-MAP (n=533) and (b) the validation dataset of brGDGT-MAP (n=179). For LSTM model, (c), The training dataset of brGDGT-MAP (n=533) and (d) the validation dataset of brGDGT-MAP (n=179). For GRU model, (e), The training dataset of brGDGT-MAP (n=533) and (f) validation dataset of brGDGT-MAP (n=179). * indicates the 99% confidence level.

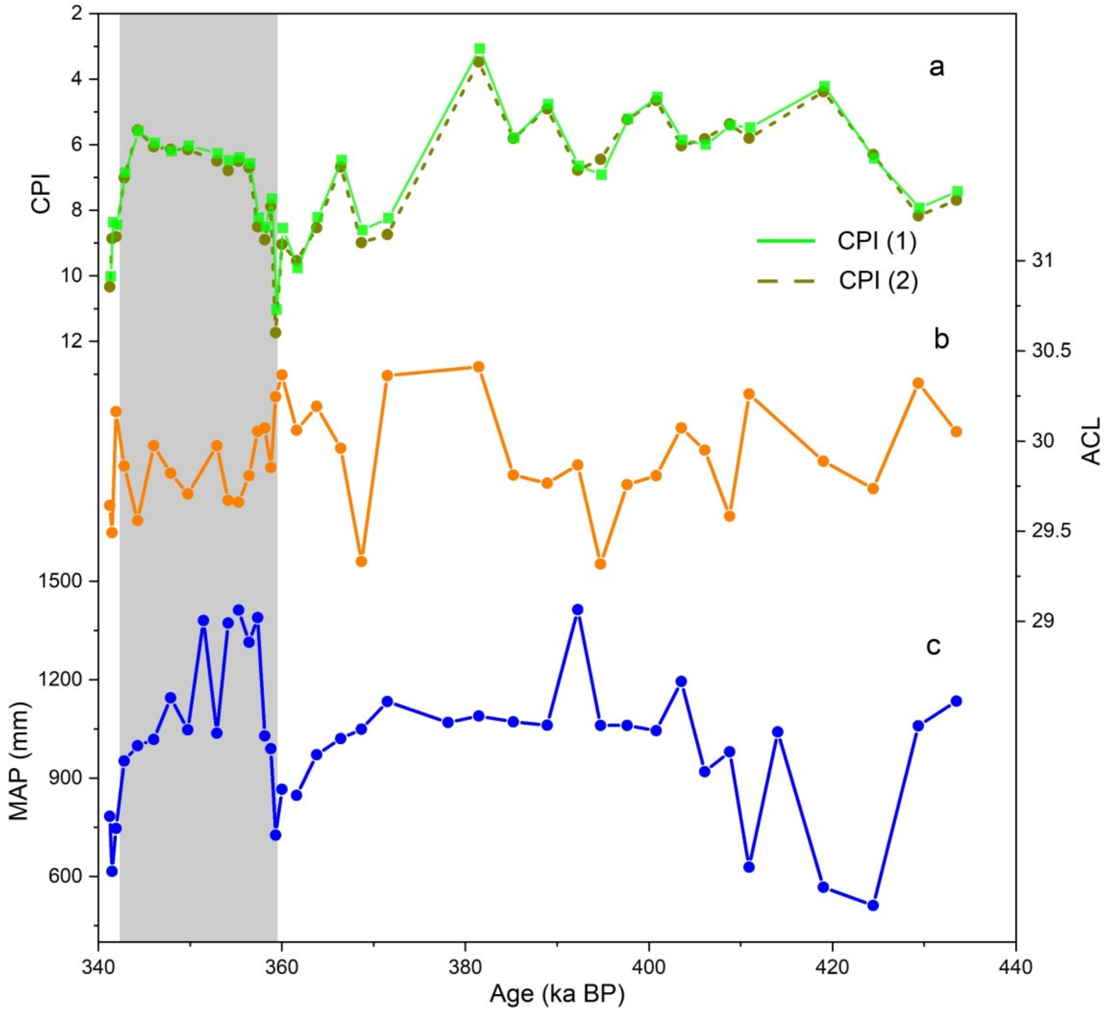

Supplementary Figure 13. Variations in MAPc we reconstructed based on brGDGTs compared with n-alkanes proxies from 340 ka BP to 430 ka BP. (a), the variations of the carbon preference index (CPI) (this study). (b), the variations of the average chain length (ACL) (this study). (c), the variations of the MAPc we reconstructed based on brGDGTs (this study).

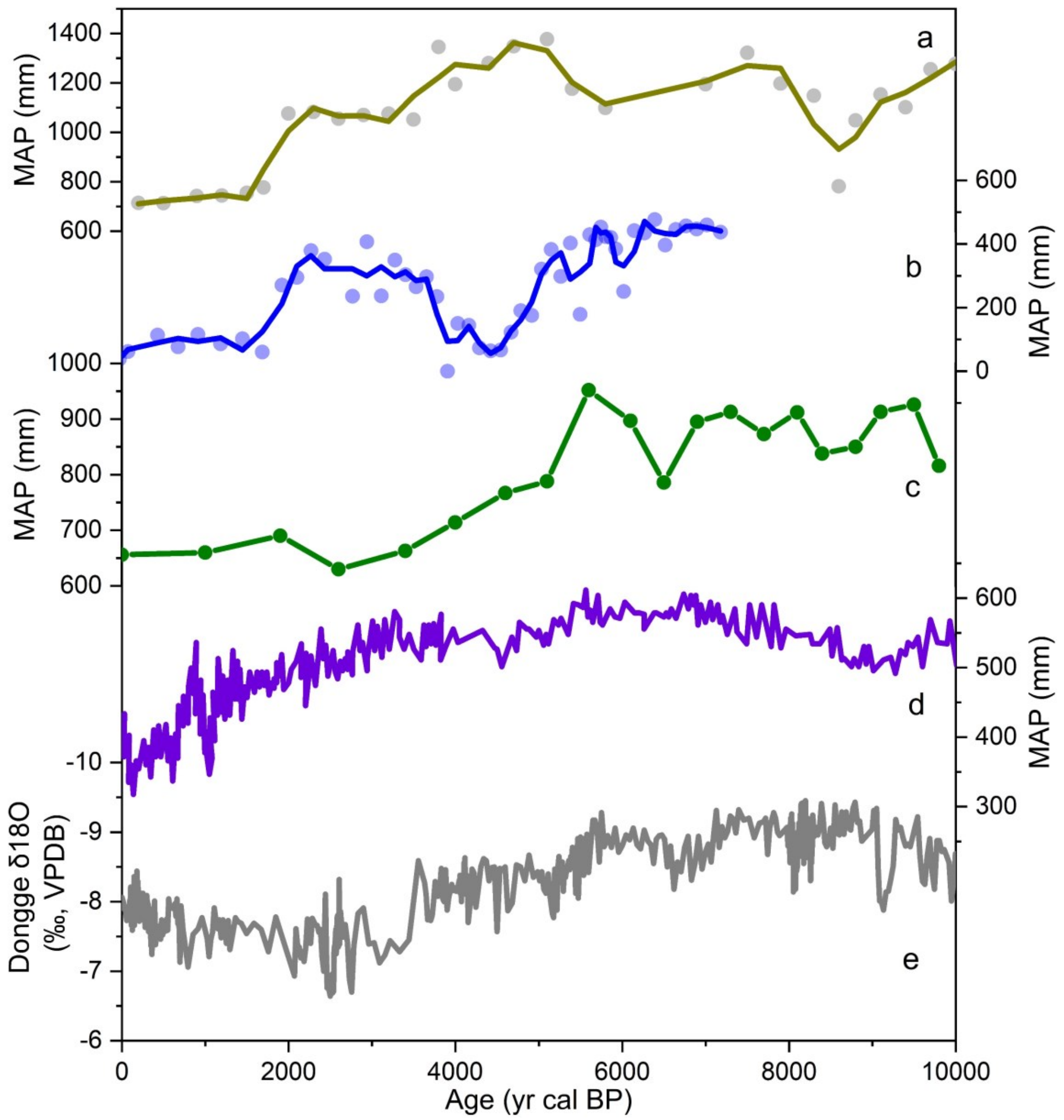

Supplementary Figure 14. The variations of MAP we reconstructed in Weinan and XRD compared with other precipitation results in EASM regions. (a), the variations of the MAP we reconstructed based on brGDGTs in Weinan LPS profile (this study, the brown solid line (five dots averaged)). (b), the variations of the MAP we reconstructed based on brGDGTs in XRD profile (this study, the blue solid line (five dots averaged)). (c), the MAP values reconstructed by ^{10}Be in Baoji LPS profile (the green solid line)[8]. (d), Annual precipitation reconstructed by a calibration function applied to the fossil pollen assemblages from Gonghai, China (the purple solid line)[57]. (e), Values of δ^{18}O from Dongge Cave (the grey solid line)[14].

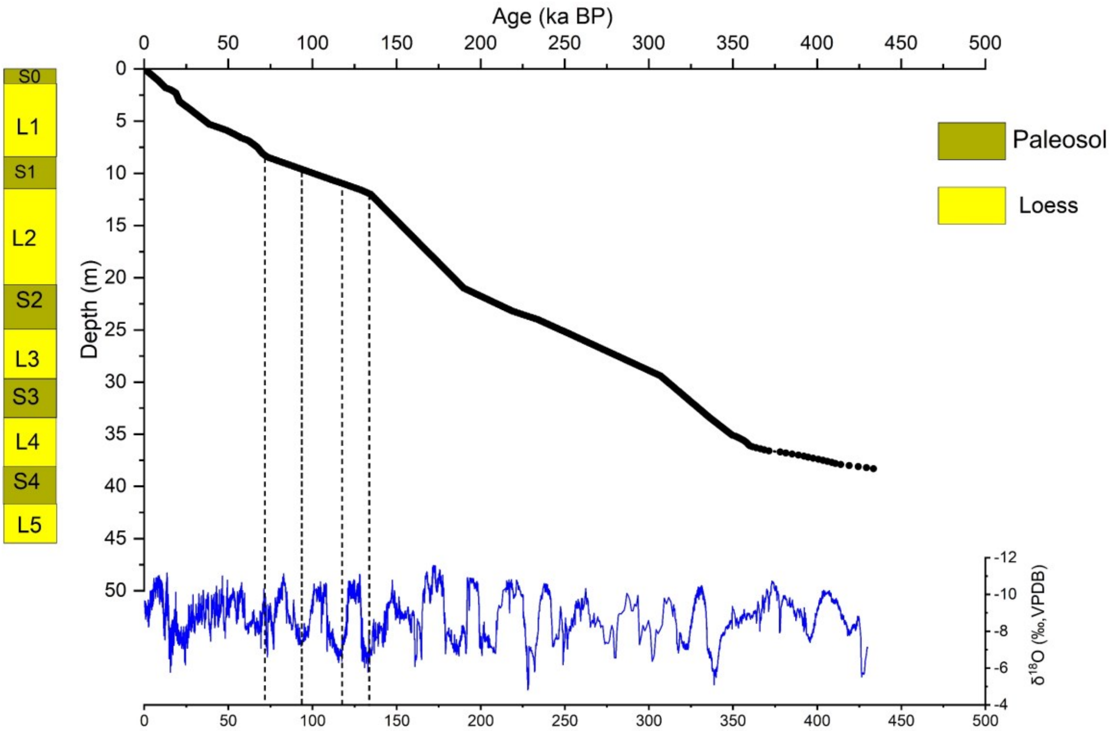

Supplementary Figure 15. Age-depth relationships picked from the Weinan profile based on 427 samples. The orange and yellow bars represent the loess-paleosol cycles in the Weinan profile.

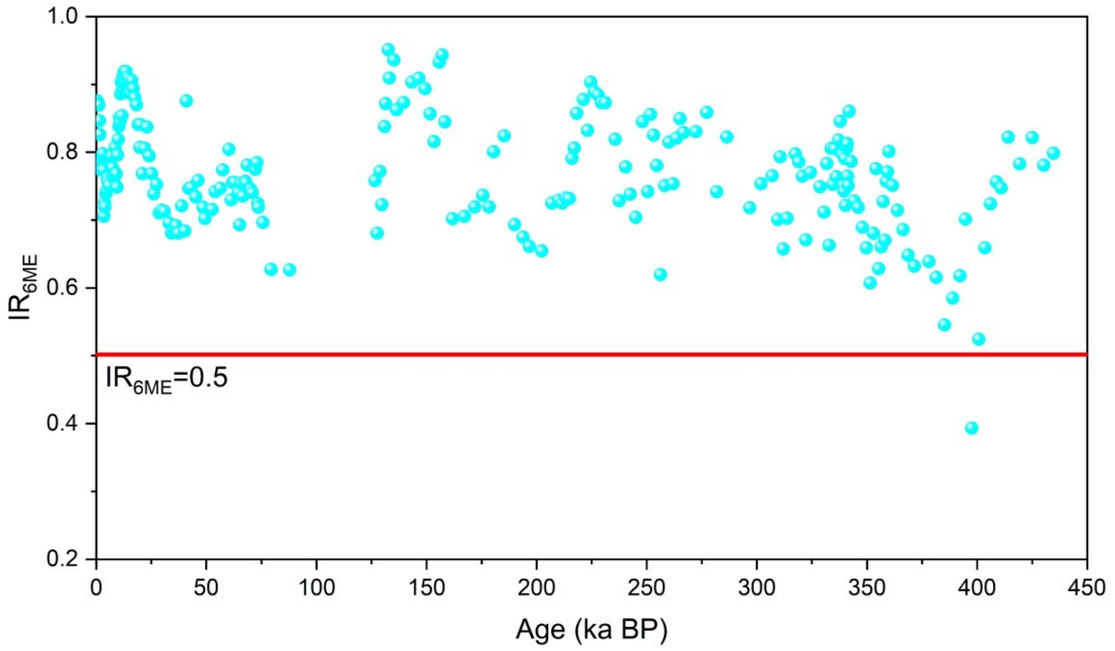

Supplementary Figure 16. Variations in the IR_{6ME} values measured in the Weinan profile over the last approximately 430 ka BP. The red solid line represents the IR_{6ME} value of 0.5.

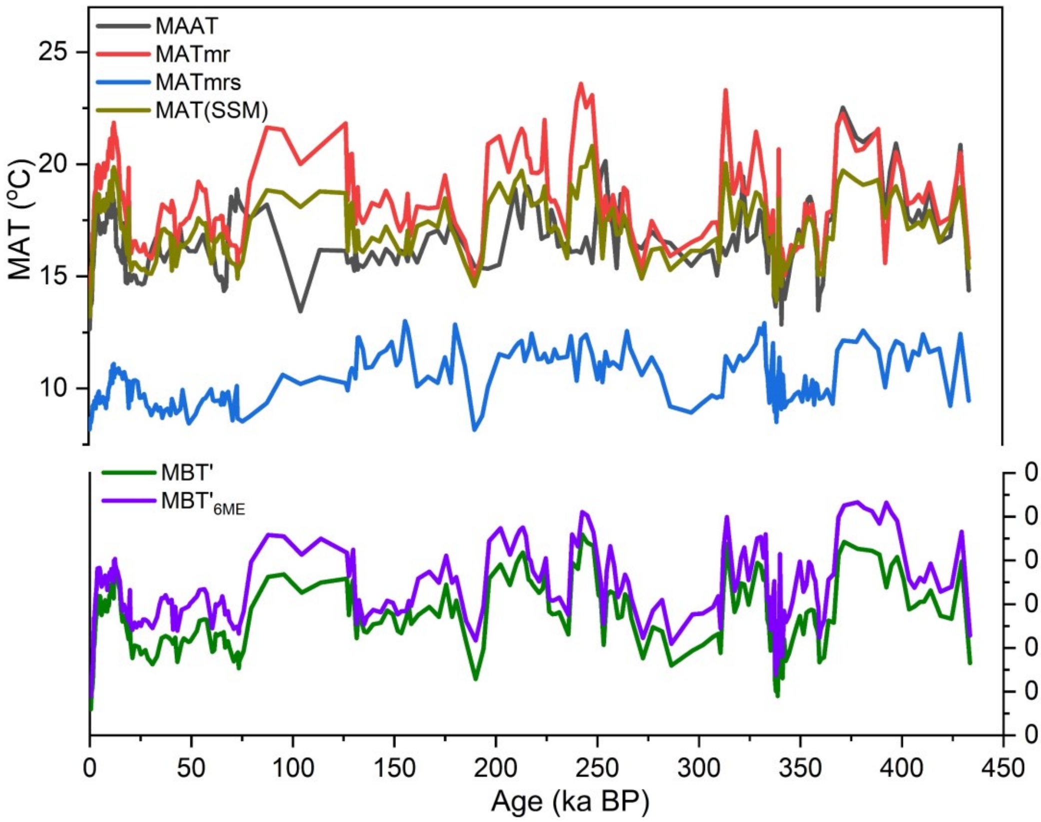

Supplementary Figure 17. The comparison of different temperature and SWC calibration models in the Weinan profile based on brGDGTs. All the references are cited in the main manuscript.

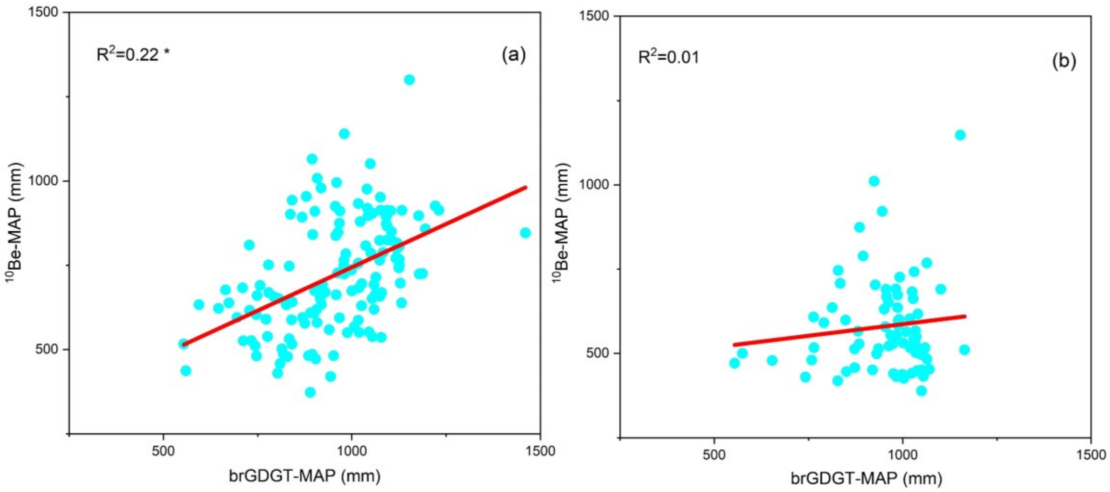

Supplementary Figure 18. The linear relationship between brGDGT-MAP and ^{10}Be-MAP. (a), Dataset containing the periods other than alternating glacial and interglacial periods. (b), Dataset containing alternating glacial and interglacial periods. * indicates the 99% confidence level.
